# Attenuation of the Hypoxia Inducible Factor Pathway after Oncolytic Adenovirus Infection Coincides with Decreased Vessel Perfusion

**DOI:** 10.3390/cancers12040851

**Published:** 2020-04-01

**Authors:** Iris Yousaf, Jakob Kaeppler, Sally Frost, Len W. Seymour, Egon J. Jacobus

**Affiliations:** 1 Anticancer Viruses and Cancer Vaccines Research Group, Department of Oncology, University of Oxford, Oxford OX3 7DQ, UK; Iris.fnu@mayo.edu (I.Y.); sally.frost@oncology.ox.ac.uk (S.F.); 2 Mechanisms of Metastasis Research Group, Department of Oncology, University of Oxford, Oxford OX3 7DQ, UK; jakob.kaeppler@oncology.ox.ac.uk

**Keywords:** adenovirus, enadenotucirev, oncolytic, hypoxia, angiogenesis, HIF, intravital imaging, two-photon microscopy, UnaG, tumor microenvironment

## Abstract

The interplay between oncolytic virus infection and tumour hypoxia is particularly unexplored in vivo, although hypoxia is present in virtually all solid carcinomas. In this study, oncolytic adenovirus infection foci were found within pimonidazole-reactive, oxygen-poor areas in a colorectal xenograft tumour, where the expression of *VEGF*, a target gene of the hypoxia-inducible factor (HIF), was attenuated. We hypothesised that adenovirus infection interferes with the HIF-signalling axis in the hypoxic tumour niche, possibly modifying the local vascular supply. In vitro, enadenotucirev (EnAd), adenovirus 11p and adenovirus 5 decreased the protein expression of HIF-1α only during the late phase of the viral life cycle by transcriptional down-regulation and not post-translational regulation. The decreasing HIF levels resulted in the down-regulation of angiogenic factors such as *VEGF*, coinciding with reduced endothelial tube formation but also increased T-cell activation in conditioned media transfer experiments. Using intravital microscopy, a decreased perfused vessel volume was observed in infected tumour nodules upon systemic delivery of EnAd, encoding the oxygen-independent fluorescent reporter UnaG to a tumour xenograft grown under an abdominal window chamber. We conclude that the attenuation of the HIF pathway upon adenoviral infection may contribute to anti-vascular and immunostimulatory effects in the periphery of established infection foci in vivo.

## 1. Introduction

Adenoviruses have been used extensively in oncolytic virotherapy and many candidates have been taken into clinical development [1,2]. The clinical efficacy of oncolytic virotherapy relies on the ability of viruses to disseminate successfully through the tumour bed, thereby maximising tumour-specific lysis, virus-mediated anti-cancer immunostimulation and the therapeutic effects of virus-encoded payloads. Understanding the local microenvironment of virus infection during treatment is essential to enable better design and improve oncolytic viral platforms. Although tumour hypoxia is a feature present in virtually all solid carcinomas, the interplay between adenovirus infection and tumour hypoxia is particularly unexplored in vivo.

The high proliferation and metabolic rate of cancer cells leaves a subset of them exposed to low levels of oxygen and nutrients. This, aggravated by the inefficient vascular supply triggered by aberrant signalling, limits the efficacy of standard anti-cancer therapies such as chemo- and radiotherapies [3,4]. While the activity of oncolytic viruses (OVs) does not rely on efficient tumour vasculature as viral progeny can disseminate from cell to cell, the role of hypoxia in anti-cancer virotherapy is less clear. The localisation of virus infection foci within hypoxic regions in vivo has only been demonstrated in the case of herpes simplex virus 1 and vesicular stomatitis virus [5,6], and it is still unclear for most OVs whether oxygen tensions affect the viral tropism and dissemination, and to what extent virus infection alters the architecture of the tumour microenvironment.

The hypoxic phenotype is predominantly driven by hypoxia-inducible factors (HIFs), which induce the transcription of many genes with tumour-promoting functions, including genes related to angiogenesis (e.g., *VEGF*, *PIGF*, *EPO*), pH homeostasis (e.g., *CAIX*), glycolysis (e.g., *GLUT1*, *PDK1*), migration, the extracellular matrix and iron transport [7,8,9,10]. These transcription factors consist of an oxygen-regulated α-subunit (HIF-1α, HIF-2α, or HIF-3α) and a constitutively expressed β-subunit (HIF-1β) [11,12]. In the presence of oxygen, the α-subunit is hydroxylated by oxygen-sensing prolyl-hydroxylases (PHDs) [13,14]. The hydroxylation leads to binding of the α-subunit by the E3 ligase and tumour suppressor von-Hippel–Lindau (VHL) resulting in its ubiquitination and proteasomal degradation [15,16]. In hypoxia, the α-subunit is not hydroxylated and heterodimerises with HIF-1β triggering the nuclear translocation of the transcription factor complex, where it binds to promoter regions containing hypoxia response elements, thereby inducing hypoxia-specific gene expression. HIF up-regulation has been linked to the viral latency regulation of several oncogenic viruses (e.g., Kaposi’s sarcoma-associated herpesvirus), which induce HIF-1α accumulation independently of the oxygen tension by altering either HIF transcription, translation, stabilisation or its transactivation activity [17]. In turn, OVs such as Newcastle disease virus, reovirus and parvovirus H1, down-regulate HIF-1α in a proteasome-dependent fashion [18,19,20], while vaccinia virus protein C16 directly binds PHDs, resulting in the accumulation of HIF-1α [21]. The effect of infection on the HIF pathway varies between virus types and remains elusive in the case of oncolytic adenoviruses.

Early phase clinical trials with the Ad11p/Ad3-chimeric group B adenovirus Enadenotucirev (EnAd), bioselected for improved replication and oncoselectivity in epithelial cancer cells [22], showed the safe delivery to remote tumour deposits following intravenous administration [23,24]. In this study, we aim to characterise the location of EnAd infection relative to hypoxic regions and define its potential influence on the hypoxic tumour microenvironment. We found that EnAd infection foci are associated with well- and poorly-oxygenated areas after intravenous injection into mice bearing colorectal xenograft tumours. In hypoxic areas, infection appeared to suppress the expression of the HIF target gene *VEGF*. We hypothesise that adenovirus infection interferes with the HIF-signalling in the hypoxic tumour niche, possibly modifying the local vascular supply and immunostimulation. In fact, adenoviral infection led to the transcriptional down-regulation of *HIF-1α* and the reduction of hypoxia-specific gene expression during the late phase of viral of infection. This coincided with improved T-cell activation and, at the same time, with the decreased ability to induce endothelial cell tube formation, and reduced vessel perfusion of infected tumour nodules in vivo.

## 2. Results

**Figure 1:**
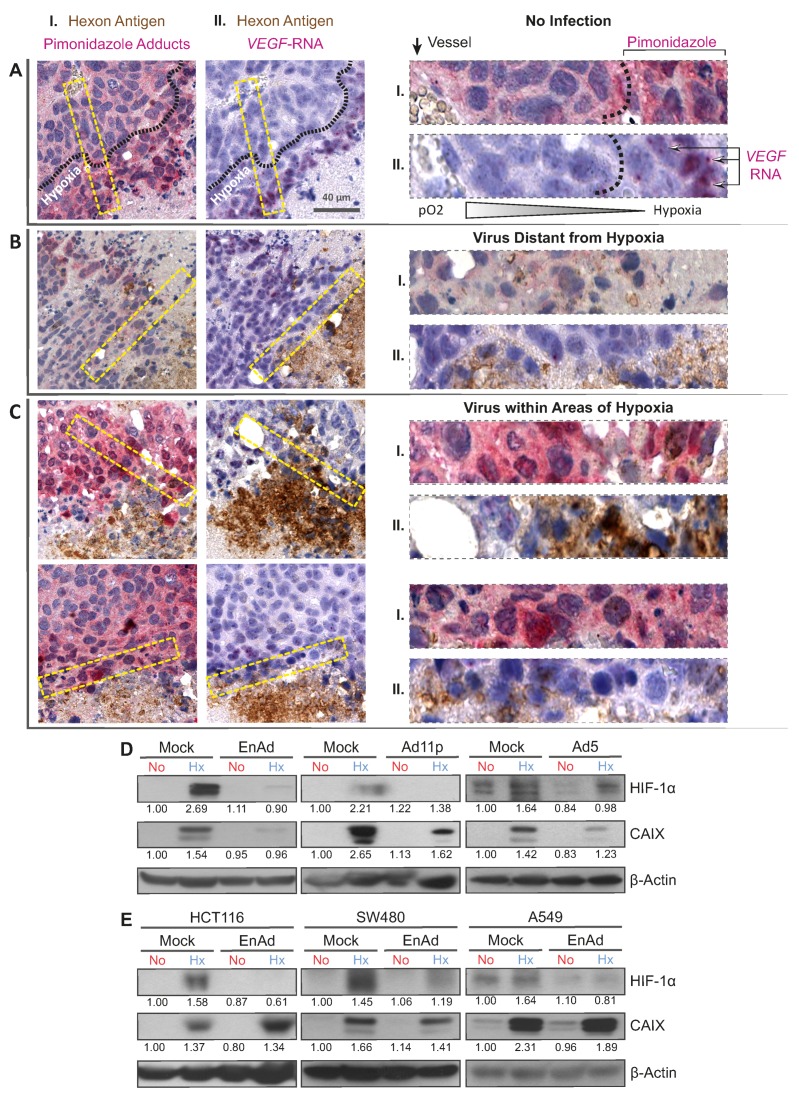
Infection foci proximal to hypoxic areas are associated with low vascular endothelial growth factor (*VEGF*) expression while adenovirus infection down-regulates HIF-1α in vitro. Hypoxic areas in DLD-1 xenograft were labelled with pimonidazole 16 days after intravenous administration of EnAd. (**A**–**C**) Serial sections were stained for Hexon (brown) and either pimonidazole adducts (I, red) or *VEGF* RNA (II, red puncta). A non-infected area (**A**), an area containing a virus distant to hypoxia (**B**) and two virus foci proximal to hypoxia (**C**) are shown. One 40 µm scale bar is provided for guidance and magnifications correspond to areas in yellow-dotted lines for staining I and II. (**D**) HIF-1α and CAIX protein expression was measured in DLD-1 cells pre-exposed to 1% pO2 (Hx, hypoxia) or 21% pO2 (No, normoxia) for 18 h followed by a 24 hour-infection with EnAd, Ad11p, and Ad5 at MOI 5. (**E**) With the same experimental setup, the down-regulation of HIF signalling was confirmed in HCT116, SW480 and A549 cells infected with EnAd at MOI 5. The corresponding unprocessed western blot images can be found in Figures S3 and S4.

**Figure A1:**
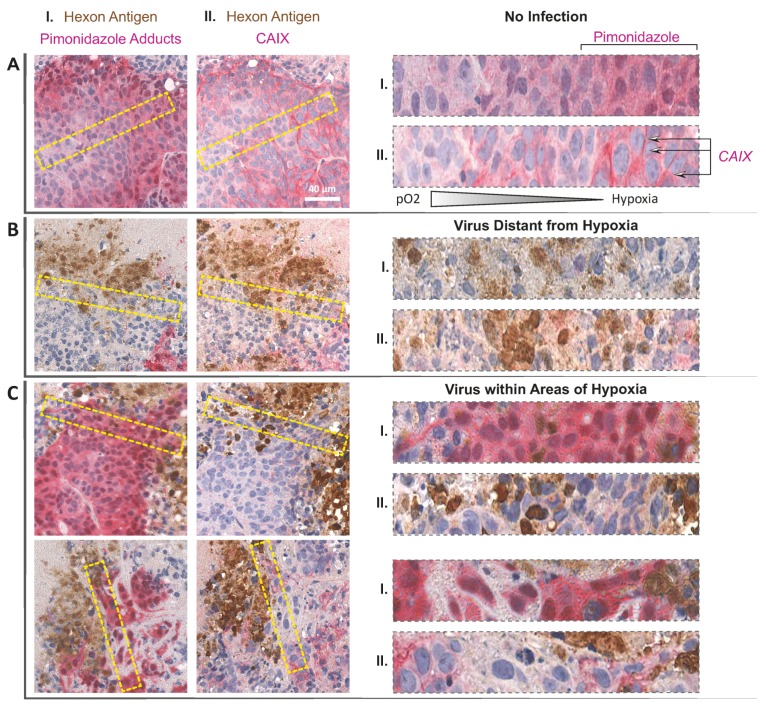
Infection foci proximal to hypoxic areas are associated with low CAIX expression. Hypoxic areas in DLD-1 xenograft were labelled with pimonidazole 16 days after intravenous administration of EnAd. (**A–C**) Serial sections were stained in brown for Hexon and in red for either pimonidazole adducts (staining I) or CAIX (staining II). A non-infected area (**A**), an area containing a virus distant to hypoxia (**B**) and two virus foci proximal to hypoxia (**C**) are shown. One 40 µm scale bar is provided for guidance and magnifications correspond to areas in yellow-dotted lines for stainings I and II. Note that CAIX membrane staining is reduced in when virus infection is found in pimonidazole-positive areas.

We investigated the location of infection sites relative to areas of hypoxia upon intravenous administration of EnAd using histological analysis. To this end, mice bearing DLD-1 xenograft tumours were treated with a fractionated dose of 4 × 10^10^ EnAd virus particles encoding the firefly luciferase gene under the major-late promoter. Once the virus replication reached a steady-state, determined by bioluminescence imaging (Figure S1), hypoxia was labelled by the administration of pimonidazole, a nitroimidazole that reacts with peptide bonds only at low levels of oxygen (<1.3%) forming immune-detectable adducts [25]. We costained serial sections obtained from tumour tissue for the viral capsid protein Hexon, detected in brown, and hypoxia-specific pimonidazole adducts, detected in pink (dual-staining I, Figure 1A–C). Established infection foci often consisted of a core of Hexon staining associated with necrotic or acellular tissue, presumably as a result of virus-induced cell death, and a rim of Hexon-positive viable tumour cells, shown by round haematoxylin-positive nuclei, representing the active infectious front (for staining controls refer to Figure S2). EnAd infection foci were found in both pimonidazole-negative (Figure 1B) and in pimonidazole-positive areas (hypoxic, Figure 1C). To further characterise the microenvironment of infection foci in the hypoxic niche, we costained serial sections for Hexon protein and *Vascular Endothelial Growth Factor* RNA (*VEGF)*, as HIF-induced *VEGF* expression is a hallmark of tissues experiencing low oxygen. In situ hybridization of *VEGF* RNA was detected as pink staining, often shown as dense puncta adjacent to cell nuclei reminiscent of polysome-associated mRNA (dual staining II, Figure 1A–C). In the absence of infection (Figure 1A), the side-by-side comparison of dual-stained serial sections showed that VEGF RNA staining overlaps with intense pimonidazole staining and they are confined to regions distant from the blood vessels, as expected in tissues experiencing hypoxia. Consequently, *VEGF* staining was absent in a control region displaying an infection focus located in a well-oxygenated pimonidazole-negative area (Figure 1B). The periphery of virus infection associated with pimonidazole-positive areas of hypoxia, however, exhibited less *VEGF* RNA staining and associated puncta (Figure 1C) than in hypoxic areas lacking virus infection (Figure 1A). In a similar histological analysis of infection foci associated to hypoxic areas, we observed decreased expression of Carbonic Anhydrase IX (CAIX, Appendix A Figure A1), another cellular biomarker of hypoxia and HIF target. This raises the possibility that virus infection leads to the decreased expression of VEGF and CAIX.

Given that *VEGF* and *CAIX* are primarily induced by HIFs, adenovirus infection may interfere with the HIF signalling axis, thereby lowering the levels of HIF target genes. To test this in vitro, we pre-incubated cancer cells under hypoxic (1% pO_2_) and normoxic (21% pO_2_) conditions, infected them at a multiplicity of infection (MOI) of five for a further 24 h, keeping the oxygen tensions constant. In all mock-infected controls, exposure to hypoxia led to the robust accumulation of HIF-1α in DLD-1 cells, while the infection of hypoxic cells with EnAd resulted in decreased expression levels of HIF-1α and its target *CAIX* (Figure 1D). This was also the case during infection with adenovirus serotype 11p (Ad11p)—the main parental virus of EnAd with 99.31% sequence homology—and the group C adenovirus serotype 5 (Ad5). This effect depended on the virus dose, suggesting that the down-regulation of HIF-1α actually occurred in the infected cells, thereby excluding the possibility of a paracrine effect in the non-infected fraction of the culture (Figure S5). The viral-induced down-regulation of HIF-1α was confirmed in HCT-116, SW480 and A549 cells (Figure 1E).

**Figure 2:**
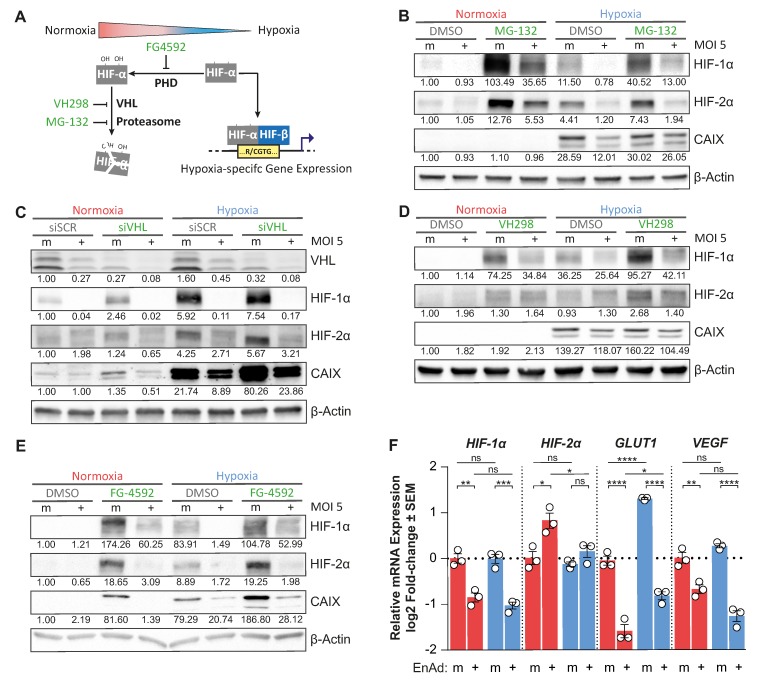
Down-regulation of HIF-1α and HIF-2α is independent of proteasomal, prolyl-hydroxylases (PHD) and von-Hippel–Lindau (VHL) activity but dependent on mRNA availability. (**A**) Schematic of the HIF-pathway indicating targets for chemical inhibition. (**B**) DLD-1 cells were pre-exposed to hypoxia (blue) or normoxia (red) for 18 h and infected with EnAd at a multiplicity of infection (MOI) of 5. The infection continued in hypoxia or normoxia for a further 24 h before western blot analysis. Proteasomal inhibition was achieved by adding 5 µM MG-132 six hours before harvest. (**C**,**D**) Inhibition of VHL was achieved by the silencing of VHL with siRNA transfection 48 h before infection, or VH298 treatment (100 µM) eight hours before harvest. (**E**) PHD inhibition was achieved by adding 75 µM FG-4592 two hours after infection. (**F**) Using the same hypoxia exposure, the mRNA expression of HIF-1α, HIF-2α, GLUT1 and VEGF was measured by RT-qPCR 24 h after infection (m: mock, +: infected, *n* = 3, ANOVA with Tukey post hoc test, ns: not significant, * *p* ≤ 0.05, ** *p* ≤ 0.01, *** *p* ≤ 0.001, **** *p* ≤ 0.0001). The corresponding unprocessed western blot images can be found in Figure S6.

The PHD-mediated hydroxylation of HIFs, which leads to its ubiquitination by VHL and proteasomal degradation, is the main pathway regulating HIF protein stability and the concomitant expression of HIF target genes in hypoxia (Figure 2A). Here, we undertook a stepwise analysis of this pathway to investigate whether virus infection alters the stability of HIF-1α and HIF-2α, since the latter isoform was also down-regulated upon virus infection, but to a lesser extent (Figure 2B–E). First, we tested the ability of EnAd to down-regulate both isoforms in the presence of the proteasomal inhibitor MG-132 in DLD-1 cells. Although six hours of MG-132 treatment before sample collection resulted in a strong stabilisation and accumulation of HIF-1α and HIF-2α in normoxic and hypoxic conditions, it did not affect the ability of the virus to down-regulate both HIF isoforms (Figure 2B). Virus-induced HIF down-regulation was neither VHL-dependent, as the inhibition of VHL by treatment with VH298 or siRNA knockdown did not restore HIF levels in infected cells (Figure 2C,D). In fact, VHL expression decreased in infected hypoxic and normoxic cells compared to uninfected controls. A lower VHL generally leads to HIF over-expression, yet HIF was found to be down-regulated in infected samples. Similarly, the addition of the PHD2 inhibitor FG-4932 (Roxadustat) did not abrogate the virally induced down-regulation of HIF-1α and HIF-2α, even though it led to the over-expression of both isoforms in hypoxic and normoxic mock-infected cells (Figure 2E). These findings were consistent in experiments with HCT116 cells (Figures S7 and S8). Taken together, the chemical inhibition of the proteasome, VHL and PHD did not restore HIF levels in infected cells, indicating that the observed HIF down-regulation is independent of HIF protein stability.

**Figure A2:**
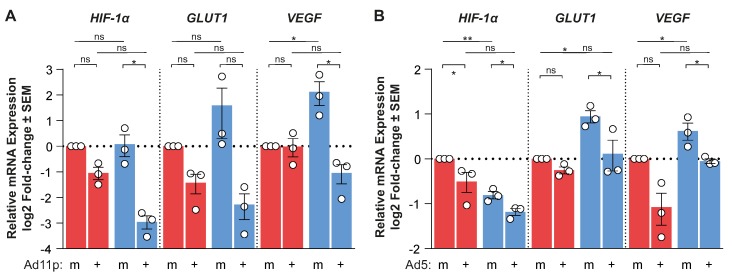
Adenovirus serotype 11p and adenovirus serotype 5 suppress mRNA levels of HIF-1α and HIF-target genes. DLD-1 cells were pre-exposed to hypoxia or normoxia for 18 h and infected with Ad11p (**A**) and Ad5 (**B**) at MOI of 5. The infection continued in hypoxia (blue) or normoxia (red) for a further 24 h. RT-qPCR was performed to measure HIF-1α, HIF-2α, GLUT1 and VEGF (m: mock, +: infected, three biological replicates, ANOVA with Tukey post hoc test, ns: not significant, * *p* ≤ 0.05, ** *p* ≤ 0.01, *** *p* ≤ 0.001).

Hence, we evaluated the possibility that adenovirus infection modulates the availability of HIF mRNA. We measured the mRNA expression of *HIF-1α*, *HIF-2α,* and the HIF-responsive genes *GLUT1* and *VEGF* by qPCR (Figure 2F). EnAd infection significantly decreased *HIF-1α* mRNA expression in hypoxia and normoxia, which correlated with decreased *GLUT1* and *VEGF* mRNA expression compared to mock-infected controls at 24 h post-infection. This transcriptional down-regulation was also observed in the case of Ad11p and Ad5 (Appendix A Figure A2). Conversely, *HIF-2α* mRNA expression was either up-regulated or unchanged in EnAd infected cells compared to mock-infected cells in normoxia and hypoxia at 24 h post-infection, respectively.

**Figure 3:**
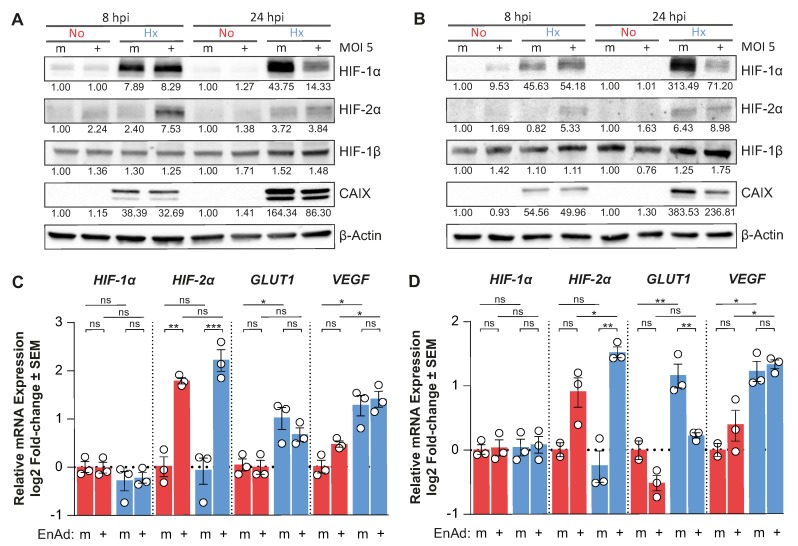
HIF expression is transiently up-regulated early and down-regulated late during viral infection. (**A**) DLD-1 cells and (**B**) HCT116 cells were pre-exposed for 18 h in normoxia (red) or hypoxia (blue) before infection with EnAd at MOI 5. The infection continued in hypoxia or normoxia for a further eight or 24 h when lysates were collected and probed for HIF-1α, HIF-2α, HIF-1β, CAIX and β-Actin by western blot. Using the same experimental setup, total RNA was collected at the eight hours post-infection from (**C**) DLD-1 and (**D**) HCT116 cells. RT-qPCR was performed to measure *HIF-1α*, *HIF-2α*, *GLUT1* and *VEGF* (m: mock, +: infected; *n* = 3; ANOVA with Tukey post hoc test, ns: not significant, * *p* ≤ 0.05, ** *p* ≤ 0.01, *** *p* ≤ 0.001). The corresponding unprocessed western blot images can be found in Figure S9.

**Figure A3:**
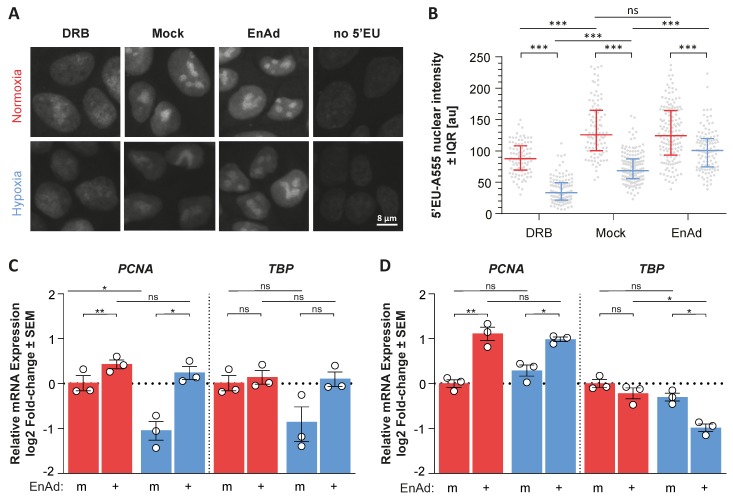
PCNA and TBP transcripts are differentially regulated during EnAd infection in hypoxia and normoxia, while the levels of global RNA synthesis are not affected by viral infection. DLD-1 cells were pre-exposed to hypoxia or normoxia for 18 h and infected with EnAd at an MOI of 5. (**A**) Global translation was measured by the incorporation of 5-ethynyl-2-uridine (5’EU, 500 µM) one hour before completion of the 24-hour infection with EnAd. Addition POL II transcription elongation inhibitor 5,6-Dichloro-1-β-D-ribofuranosyl-benzimidazole (DRB, 100 µM) two hours before 5’EU labelling served as negative control. 5’EU was detected by a click-chemistry reaction using Alexa555 fluorophore (Click-iT, Thermo Fisher) and imaged by confocal microscopy. (**B**) Each data point represents the median fluorescence intensity of 5’EU-Alexa555 in individual nuclei (*n* > 90). The fluorescence intensity corresponds to the sum projection of 0.8 µm optical sections covering the entire cell nucleus; median ± the interquartile range are shown. Samples with no 5’EU-pulse were used to subtract background signal. RT-qPCR was performed to measure *Proliferating Cell Nuclear Antigen* (*PCNA*) and *TATA-box Binding Protein* (*TBP*) mRNA expression at 8 (**C**) or 24 h (**D**) in normoxic (red) and hypoxic (blue) cultures, m: mock, +: infected, three biological replicates. Statistical significance was assessed using ANOVA with Tukey post hoc test, ns: not significant, * *p* ≤ 0.05, ** *p* ≤ 0.01, *** *p* ≤ 0.001).

The decrease in *HIF-1α* mRNA availability and concomitant down-regulation of HIF-target genes indicated that EnAd infection suppresses *HIF-1α* mRNA transcription, thereby decreasing the accumulation of HIF-1α protein during hypoxic culture. Additionally, we observed that the down-regulation of HIF expression only occured late after infection (24 h post-infection); in fact, HIF-1α, and particularly HIF-2α, were up-regulated in hypoxic DLD-1 and HCT116 cells at eight hours post-infection (Figure 3A,B). Notably, EnAd infection only altered the expression of the α-subunits, as the HIF-1β protein levels are virtually unchanged throughout infection or hypoxic treatment. At eight hours post-infection, the *HIF-1α* and *VEGF* mRNA levels in infected cells were comparable to mock controls, while EnAd infection significantly induced the mRNA expression of *HIF-2α* and reduced the levels of *GLUT1* mRNA compared to mock-infected cells (Figure 3C,D). Notably, we observed that virus infection did not decrease global RNA synthesis as measured by the incorporation of an RNA nucleotide analogue into nascent RNA chains (Appendix A Figure A3A,B). Additionally, EnAd infection led to the up- and down-regulation of host genes not directly related to the HIF-pathway such as *Proliferating Cell Nuclear Antigen* (*PCNA*) and *TATA-box Binding Protein* (*TBP*) in both hypoxic and normoxic cultures, respectively (Appendix A Figure A3C,D). This evidences that viral infection rewires the host cell transcriptional programme. In the context of hypoxic signalling, the results indicate a viral life-cycle-dependent modulation of HIF expression, where HIF-2α was transiently up-regulated early during infection followed by the down-regulation of mainly HIF-1α late during infection, which largely influence hypoxia-specific gene induction.

**Figure 4:**
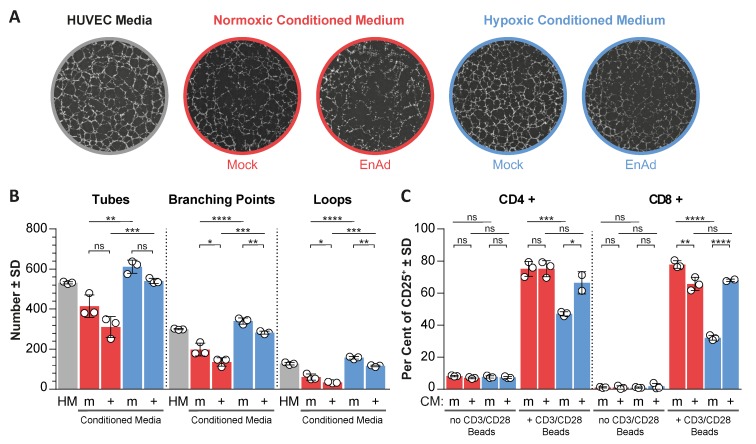
Conditioned media from adenovirus-infected cell cultures reduces the angiogenic potential of human umbilical vein endothelial cells (HUVEC) and increases T-cell activation. Conditioned media (CM) from DLD-1 cancer cells, pre-exposed to 18 h of normoxia (red) or hypoxia (blue) followed by a further 24 h of mock (m) or EnAd infection at MOI 5 (+), were collected and cleared from debris and infectious virus particles (Figure S10) (**A**) Tube formation assay: CM and HUVEC growth media containing VEGF (HM) were incubated with HUVECs on Matrigel to allow endothelial cells to form vessel-like structures; representative images are shown. (**B**) The angiogenic potential of the CM was assessed by measuring the number of tubes, branching points and loops (space surrounded by segments). (**C**) T-cell activation assay: A coculture of CD3-positive lymphocytes isolated from peripheral blood and DLD-1 cancer cells (at a ratio of five to one) was exposed to CM. T-cell activation was induced using CD3/CD28 T-cell activation beads. The percentage of CD25-positive CD4 and CD8 T-cells was measured as illustrated in the Figure S11. Statistical significance was tested by two-way ANOVA with Tukey post hoc test, *n* = 3, ns: not significant, * *p* ≤ 0.05, ** *p* ≤ 0.01, *** *p* ≤ 0.001, **** *p* ≤ 0.0001.

**Figure A4:**
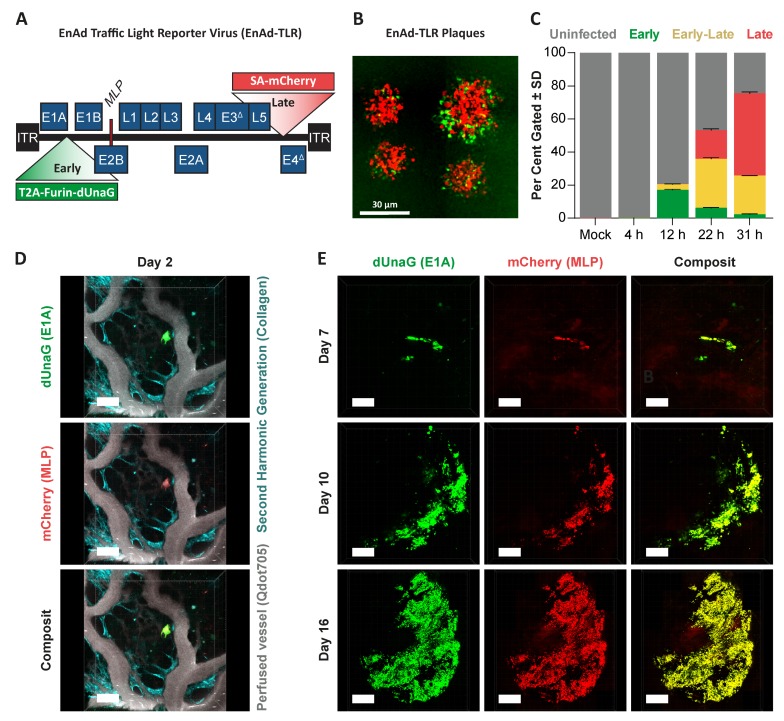
The concerted expression of early and late viral gene expression prevails in tumour xenografts using a traffic light adenovirus reporter. (**A**) Enadenotucirev (EnAd) was engineered with UnaG and mCherry to report early and late viral gene activity, respectively. Una, containing a PEST destabilising sequence for improved turnover was fused to E1A via a T2A-Furin linker. mCherry, also with a 3’PEST sequence, was cloned downstream of the fibre gene under the control of the major late promoter (as in Figure S11). (**B**) This dual reporter virus (EnAd-traffic light virus, EnAd-TLV) gave rise to plaques which had green cells at the infection front and red cells at the core. (**C**) DLD-1 cells were infected at a multiplicity of infection of 5 and harvested at different time points and analysed by flow cytometry. Uninfected, early-phase, early/late-phase and late-phase populations were identified. (**D**) A mouse with an unlabelled DLD-1 tumour grown in an abdominal window chamber received 2 × 10^10^ EnAd-TLV particles. Two days after a single infected cell was identified next to a perfused blood vessel (Qdot705). Collagen fibres were here visualised by second harmonic generation through a 436/20 bandpass filter. (**E**) In a second mouse, EnAd-TLV infection was tracked at days 7, 10 and 16. Interestingly, the bulk of the infection focus is dominated by overlapping green and red fluorescence. Scale bars represent 250 µm.

Using an adenovirus reporting early and late viral activity (EnAd traffic light reporter), we showed that viral late gene expression predominated over viral early gene expression in a single-step infection in DLD-1 cells (Appendix A Figure A4A–C). Consequently, in solid xenograft tumours, most infected cells showed concerted early and late viral gene expression (Appendix A Figure A4D,E), indicating that during steady-state infection, a late-phase phenotype may dominate. This implied a net suppression of HIF signalling and hypoxia gene expression, as HIF down-regulation was only observed late after infection. Hence, we considered the possibility that the suppression of angiogenic factors, such as VEGF in response to virus-induced HIF down-regulation, could have an impact on the tumour microenvironment by altering the angiogenic potential of endothelial cells and activation of T-cells, as both are affected by the presence of VEGF [26,27,28]. We tested the ability of human umbilical vein endothelial cells (HUVEC) cells to form vascular-like networks (tube formation) in the presence of conditioned media from mock and virus-infected cells exposed to normoxic and hypoxic conditions, where conditioned media were cleared from live virus particles (Figure S10). Three parameters were quantified, namely the number of tubes, the number of branching points and the number of loops (spaces surrounded by tubes in all directions) (Figure 4), whereas an increase in these values correlates with increased angiogenic potential. Conditioned media from hypoxic uninfected cells significantly increased all three parameters compared to normoxic controls, while conditioned media from infected normoxic and hypoxic cells led to a decrease in all three parameters compared to uninfected controls. On the other hand, hypoxic-conditioned media significantly reduced the activation of T-cells, based on the expression of the activation marker CD25, compared to normoxic-conditioned media. Notably, conditioned media from EnAd-infected hypoxic cancer cells partially rescued this effect. The increase in T-cell activation and reduced angiogenic potential of HUVECs in the presence of conditioned media from infected hypoxic cultures coincided with reduced HIF signalling upon infection, suggesting that EnAd could exert similar effects in vivo.

**Figure A5:**
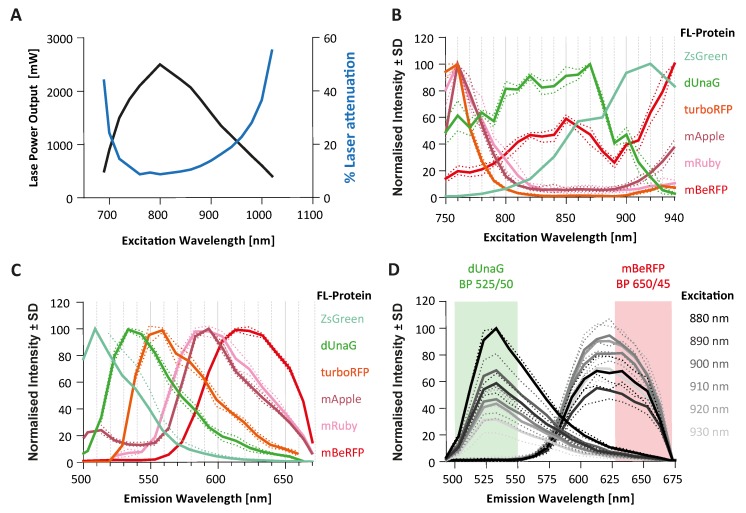
The oxygen-independent fluorescent protein dUnaG is best matched with mBeRFP for multiphoton imaging. Optical characterisation and selection of fluorescent reporters for visualisation of tumour cells and virus multiphoton imaging (MPI). (**A**) The per cent attenuation of laser power to ensure equal excitation of different wavelengths was calculated by measuring the laser power output with a galvanometer at different wavelengths. (**B**) Emission intensities of different fluorescent proteins were acquired at different excitation wavelengths. (**C**) Emission spectra of DLD-1 cells transfected with plasmids encoding different fluorescent proteins was acquired with optimal multiphoton excitation. The intensity was normalised to the emission maxima, *n* = 5 regions of interest. (**D**) The emission intensity of cells transfected with both dUnaG and mBeRFP were acquired at different excitation wavelengths using a spectral detector. Bandpass filters for adequate for dUnaG and mBeRFP two-photon acquisition with an external detector were overlaid on the emission spectra.

We next asked whether EnAd infection can directly influence the surrounding tumour microenvironment by altering the tumour vasculature at the functional level. We set out to visualise the tumour vasculature by live two-photon imaging of a fluorescently labelled subcutaneous tumour implanted within an abdominal window chamber during treatment with EnAd encoding a fluorescent reporter. To this end, we first measured the fluorescence emission of an array of green, red and far-red fluorescent proteins (ZsGreen, UnaG, turboRFP, mApple, mRuby, and mBeRFP) for their use in two-photon microscopy across wavelengths between 750 to 940 nm while maintaining a constant laser power output (Appendix A Figure A5A–C). Only ZsGreen, UnaG and mBeRFP were excited within 870 to 940 nm, a near-infrared illumination range that mitigates photodamage and ensures light penetration into the tissue without exhausting the laser power output, thus making them suitable for in vivo imaging. Even though ZsGreen showed a better excitation profile at longer wavelengths than UnaG, the fluorescence emission of UnaG is oxygen-independent, and thus better suited to visualise virus dissemination in areas that may be hypoxic [29,30]. Hence, we engineered EnAd to express a replication-dependent and destabilised version of UnaG with a reduced half-life (EnAd-SA-dUnaG) to report virus infection (Figure S12). mBeRFP under the control of a CMV promoter, was then used to label DLD-1 cancer cells via lentiviral transduction (Figure S13). Within an excitation range of 880 to 930 nm, shorter wavelengths favoured the excitation of dUnaG, while longer wavelengths favoured the excitation of mBeRFP (Appendix A Figure A5D). The strong expression levels of the virally encoded dUnaG, compared with the expression of mBeRFP by the cancer cells, allowed us to image both fluorescent proteins and a fluorescent perfusion tracer (Qdot705) simultaneously using a single excitation wavelength of 910 nm. 

**Figure 5:**
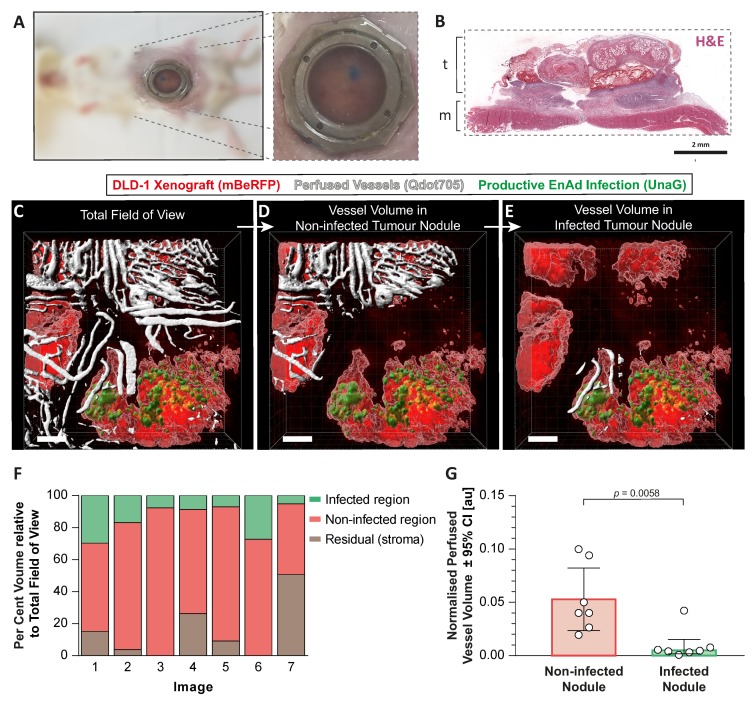
EnAd-infected tumour nodules exhibit lower vascular perfusion compared to non-infected tumour nodules. (**A**) DLD-1-mBeRFP cells loaded with Evan’s blue dye were injected above the abdominal muscle layer into the connective tissue of immunodeficient mice under an abdominal window chamber. (**B**) The location of the tumour (t) and the muscle (m) in this tumour model are shown by haematoxylin and eosin staining. (**C**) Once the mBeRFP-DLD-1tumour was established, 4×10^10^ EnAd-SA-dUnaG particles were injected intravenously. The replication-dependent expression of dUnaG (green) within the tumour (red) and perfused vascular volume (white) by the infusion of fluorescent nanocrystals is shown in a three-dimensional reconstruction of a representative image acquired by two-photon microscopy. Regions of interest comprising a non-infected tumour region (**D**) and a region containing an established infection focus (**E**) were selected based on the segmented tumour volume. (**F**) The volume percentage of infected regions (green), tumour regions (red) and non-fluorescent areas (brown, tumour stroma) relative to the whole image volume is shown for the seven infection foci acquired collectively from three mice covering a depth range of 335 to 400 µm. (**G**) Perfused vessel volumes in regions of interest as described in D and E were compared and significance was assessed by using a Mann–Whitney test. Scale bars represent 250 µm.

**Figure A6:**
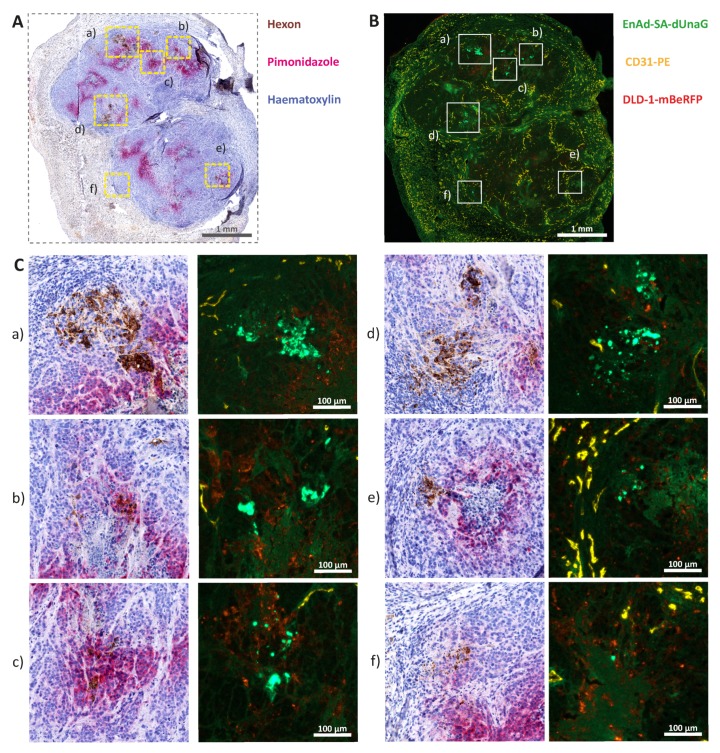
EnAd infection localised to pimonidazole positive and negative areas in tumours grown under and abdominal imaging window and ex vivo validation of EnAd-SA-dUnaG dissemination in the MPI-imaged tumour in 10 µm thick cryo-sections. (**A**) Hexon (brown) and pimonidazole adducts (red) haematoxylin (blue) positive areas were detected by immunohistochemistry. (**B**) A contiguous section was imaged by confocal spectral imaging at 488 nm. Left: The image represents true colour of the fluorescent markers used: red for tumour, bright green for EnAd and yellow for perfused vessels. The luminal side of perfused vessels was stained by injection of 50 µL of a CD31-PE antibodies. Right: The image is the result of linear unmixing and represents the specific emission of the fluorescent markers minus residual fluorescence (autofluorescence). (**C**) High magnification of matching immunohistochemistry and fluorescence images are shown for six infection foci (a–f).

An abdominal window chamber surgically implanted in CB17-SCID mice, in combination with DLD-1-mBeRFP tumour cells injected subcutaneously, enabled us to observe the growing tumour (Figure 5A,B). Once a functional vasculature developed, EnAd-SA-dUnaG was delivered intravenously. The colonisation and dissemination of the reporter virus were followed by two-photon imaging until established infection foci were detectable (up to two weeks). While the perfused tumour vasculature was labelled with a fluorescent tracer, we imaged infected and non-infected tumour foci enabling the quantification of the perfused vascular volume in these two regions of interest (Figure 5C–F). Indeed, the quantification of segmented in vivo images of infected foci showed less functional, perfused vascular volume compared to non-infected tumour foci (Figure 5G). The specificity of the UnaG signal and the relative location of infection foci to perfused vessels detected by microscopy was validated ex vivo. Whole-tissue serial slices from a resected tumour that received fluorescently labelled anti-CD31 antibodies (labelling the lumen of perfused vessels) and pimonidazole were either imaged by fluorescence microscopy or costained for EnAd particles (Hexon) and pimonidazole-adducts. A side-by-side comparison shows the colocalisation of UnaG fluorescence and hexon protein (Appendix A Figure A6). As is typically observed in tumours, the perfused blood vessel density (CD31 signal) was lower within the tumour than in the surrounding tissue, and areas lacking pimonidazole staining coincided with areas containing perfused vessels. EnAd-infected cells were again found in both pimonidazole-positive and -negative areas, as described in Figure 1. Here, we also observed evidence of EnAd replication in areas distant from perfused blood vessels, confirming the results acquired by two-photon microscopy.

## 3. Discussion

This study provides evidence that adenoviruses, when delivered systemically to solid tumour xenografts, can be found in hypoxic tumour regions and alter the local microenvironment by limiting vessel perfusion. Our in vitro models indicate that the infection of hypoxic cancer cells can reduce angiogenesis, possibly due a significant decrease in *HIF-1α* transcription in the prevailing late phase of viral infection, leading to a lower prevalence of angiogenic factors including, but perhaps not limited to, VEGF. Although, EnAd infection led to two previously described outcomes attributed to reduced VEGF levels in hypoxia, namely the decrease in endothelial tube formation and the increase in T-cell activation [26,27], histological analysis revealed reduced *VEGF* expression in infection foci within hypoxia areas. We conclude that, during therapy, adenovirus infection could contribute to a localised reduction in vascular perfusion, and perhaps even to the alleviation of immunosuppression.

Adenoviruses appear to only suppress HIF-1α at the transcriptional level, in contrast to other oncolytic viruses that alter the proteasomal pathway to destabilise HIF-1α [18,31], or, as recently described, inhibit HIF-1α activity by preventing its nuclear localisation [32]. Even though HIF activity is mainly controlled by post-translational regulation, several studies highlight the contribution of HIF-1α mRNA expression towards hypoxia-specific gene expression [33,34,35]. In our study, this was shown by a lack of HIF accumulation in virus-infected cells even when VHL was inhibited. The precise mechanism of *HIF-1α* mRNA suppression after infection remains unknown, however it is unlikely that *HIF-1α* down-regulation is a consequence of transcriptional shut-down, since *HIF-2α* levels remain unchanged in infected hypoxic and normoxic cells at 24 h post-infection. In fact, EnAd infection did not impact on the levels of global RNA synthesis and Ad5 infection has been shown to trigger global changes in gene expression, leading to both gene induction and suppression [36]. Hypoxia and adenovirus infection may decrease the rate of mRNA translation and contribute to the overall down-regulation of HIF particularly at late timepoints during infection [37], although, this is less plausible as HIF-1β, with a half-life of approximately five hours [38], is constitutively expressed throughout infection in hypoxic and normoxic cells. Interestingly, the transcription factor SP1, which is largely responsible to sustain HIF-1α expression [39], is also involved in the control of adenoviral promoters such as the major late promoter [40], raising the possibility that viral promoters and *HIF-1α* promoters may compete for this transcription factor. Furthermore, the inhibition of the nuclear factor-κB (NFκB) pathway, which can occur upon Ad5 infection [41], drastically reduces the basal *HIF-1α* mRNA production [42]; thus, it is conceivable that adenovirus may decrease HIF-1α expression indirectly by the inhibition of NFκB signalling.

The role of HIF signalling in adenovirus infection is still controversial. For example, the constitutive HIF expression in renal cancer cells defective in VHL does not correlate with sensitivity to Ad5 infection [43]. Conversely, hypoxia leads to decreased replication efficiency and oncolysis during group B and C adenovirus infections at low MOI [44,45,46]. As for other oncolytic viruses, HIF over-expression increased the expression of antiviral factors such as IFN-β, which conferred resistance to vesicular stomatitis virus infection [47], while hypoxia mimetic compounds decreased the cytotoxicity mediated by the rat parvovirus H1. However, the latter suppresses HIF-1α levels by intercepting the proteasomal pathway [20], possibly circumventing eventual HIF-related anti-viral effects. Regarding adenovirus, we identified a viral life cycle-dependent modulation of HIF expression, with a transient up-regulation of mainly HIF-2α and predominant down-regulation of HIF-1α. This finding may help in understanding the interplay between the HIF-pathway and adenovirus infection.

To our knowledge, this is the first report on the visualisation and monitoring of an oncolytic virus infection under an abdominal tumour window chamber. Compared to the more frequently used dorsal skin-fold model, the abdominal window chamber does not lead to vasoconstriction and reduces the incidence of inflammation, known to alter angiogenesis [48]. The use of mBeRFP and UnaG for intravital two-photon imaging allowed us to follow tumour growth, virus dissemination and to evaluate the impact of virus infection on the surrounding tumour microenvironment by assessing vascular perfusion. The use of an oxygen-independent fluorescent protein such as UnaG enabled the detection of virus associated with hypoxic tumour regions, which otherwise, labelled with a GFP-like fluorescent protein, may have been missed, as GFP requires oxygen for chromophore formation [49]. The importance of using an oxygen-independent reporter was also highlighted in a recent study on the visualisation of leukocytes and fluorescently tagged oncolytic viruses during the surgical exposure of live murine tissues [50].

From our histological analysis, we conclude that virus foci do not localise exclusively to well-oxygenated areas, as is widely assumed. In fact, EnAd infection in hypoxic areas was a common feature. The long-term persistence of adenovirus infection and lack of complete tumour coverage observed in xenografts models and human trials has suggested that hypoxia might hinder viral spread [23,51]. Nonetheless, hypoxia has not been proven to directly limit viral dissemination in vivo. The use of hypoxia-alleviating strategies and the further development of our intravital microscopy model, incorporating phosphorescent probes that accurately measure the oxygen tensions [52], could be instrumental to characterising the tropism of oncolytic viruses within the oxygen gradient in solid tumours and help to understand the role of hypoxia on virus dissemination.

The use of an intravenous administration route exposes oncolytic viruses initially to well-oxygenated tumour cells before potentially reaching hypoxic areas or encountering hypoxia due to tumour cell outgrowth or perfusion changes within the vascular bed. Since infected tumour nodules exhibited decreased vessel perfusion, it is plausible that viral infection could further exacerbate any oxygen deficiencies, leaving more virus foci associated with hypoxia. In this regard, we have collected molecular, histological and functional evidence indicating that the negative impact of EnAd on the HIF pathway and *VEGF* expression may contribute towards decreased vessel perfusion. Other oncolytic viruses have been shown to significantly reduce vascular supply by the direct infection and killing of activated endothelium, revealing another key axis of their anti-cancer mode of action [53,54]. It is unlikely that EnAd directly targets endothelial cells, as infection of human endothelial cells is poor and murine cells are refractory to EnAd infection [55,56]. However, it remains unclear whether the reduction in vessel perfusion observed in EnAd-infected tumour nodules contributes to its anticancer activity in vivo. Furthermore, the adenovirus-triggered suppression of *HIF* and *VEGF* expression might also contribute towards the remodelling of the local tumour immune landscape, curtailing the wide-ranged immunosuppressive effects of VEGF [57].

## 4. Materials and Methods

### 4.1. Cells and Compounds

DLD-1, HCT-116, SW-480, A549 (American Type Culture Collection) and AD293 cells (Agilent, Santa Clara, CA, USA) were cultured and grown in DMEM supplemented with 10% heat-inactivated foetal bovine serum (Thermo, Abingdon, UK). Human vein endothelial cells were cultured in EGM-2 complete medium (Lonza, Walkersville, MD, USA). Short tandem repeat profiling (Source Bioscience, Nottingham, UK) and mycoplasma testing (Lonza, Walkersville, MD, USA) were performed routinely. MG-132, FG-4592, 5,6-Dichloro-1-β-D-ribofuranosyl-benzimidazole (DRB) were purchased from Sigma Aldrich (Gillingham, UK), VH298 from Abcam (Cambridge, UK), Pimonidazole from Hypoxyprobe (Burlington, MA, USA) and 5-ethynyl-2-uridine (5’EU) from Jena Bioscience (Jena, Germany).

### 4.2. Viruses

EnAd-SA-FLuc, here referred to as EnAd, contains a Firefly luciferase coupled to the major late promoter by a splice acceptor sequence (seed stock provided to Psioxus Therapeutics, Oxford, UK) [58]. Ad5-E1A-FLuc, here referred to as Ad5, is a replication-competent reporter adenovirus that encodes firefly luciferase fused to E1A protein [59]. Ad11 is a wildtype Ad11p virus. The generation of EnAd-SA-dUnaG and EnAd-traffic light reporter virus (EnAd-TLR) is illustrated in the Supplementary Figure S12 and in the Appendix A Figure A4, respectively. All virus stocks were concentrated and purified by a caesium chloride density gradient and titred by plaque assay on A549 cells as previously described [60].

### 4.3. Hypoxia

Hypoxia incubation was carried out at 1% pO_2_ using the in vivo 2400 chamber (Baker Ruskin, Bridgend, UK). The treatment was initiated by replacing the culture medium that was equilibrated overnight at 1% pO_2_. To maintain equilibrated oxygen tensions, plastic culture dishes were placed on a side-to-side rocker at five cycles per minute (PMR-30, VWR). External oxygen calibration was performed routinely.

### 4.4. In Vivo Hypoxia Labelling

Six to eight-week-old female CB17-SCID mice were inoculated with 2.5 × 10^6^ DLD-1 cells on the right flank. Mice bearing xenografts between 80–120 mm^3^ were treated with 4 × 10^10^ EnAd-SA-FLuc particles via the tail vein in two doses on days one and three. Sixteen days after treatment, mice received an intraperitoneal injection of pimonidazole at 60 mg·kg^−1^. Two hours afterwards, animals were culled and tumours were explanted for histological analysis.

### 4.5. Immunohistochemistry and In Situ Hybridization

Tumours were fixed in 10% normal-buffered formalin prior to paraffin embedding (Leica tissue processor). Antibody staining was carried out with the automated Leica Bond Max (Leica, Milton Keynes, UK). Four-micron tissue slices were first incubated with anti-Hexon antibody (1:300 dilution, 1 h, clone 20/11, Merck Millipore, Watford, UK) and developed with a brown chromogen. Subsequently, epitope retrieval was performed at 60 °C for 20 min using Epitope Retrieval solution 2, followed by incubation with an anti-pimonidazole antibody (1:500 dilution, 30 min, P2627, Hypoxyprobe, Burlington, MA, USA) and red chromogen detection. For dual staining of *VEGF* RNA and Hexon antigen, the VEGF probe (#423161, ACD, Newark, CA, USA) was first hybridized and developed with a red chromogen according to the manufacturer’s protocol. Mouse IgG1 isotype (ab37355) and rabbit IgG isotype (ab171870) were used for staining controls (Abcam, Cambridge, UK). Slides were then stained for Hexon antigen as described above. Tumours were counterstained with haematoxylin, mounted and imaged with the Aperio Imagescope (Leica, Milton Keynes, UK).

### 4.6. Immunoblotting

Monolayers were harvested in normoxia or hypoxia in 80 μL of RIPA buffer supplemented with 3× Halt protease and phosphatase inhibitor (Thermo, Abingdon, UK). Lysates were homogenised using a Branson digital sonifier for 15 s at 10% power on ice. Supernatants were cleared from debris by centrifugation at 25,000 *g* for 20 min at 4 °C. Protein concentrations were measured using a bicinchoninic acid assay (Pierce). A range from 20 to 50 µg of denatured protein samples in Laemmli buffer (Alfa Aesar, Heysham, UK) was separated in 4% to 20% sodium dodecyl sulphate-polyacrylamide gels and transferred by electroblotting onto nitrocellulose membranes (Biorad, Watford, UK). Primary antibodies were anti-HIF-1α (clone 54, BD Biosciences), anti-HIF-2α (clone D9E3), VHL (polyclonal, Cell Signalling, Beverly, MA, US), anti-CAIX (clone M75, Biosciences Slovakia, Bratislava, Slovakia), and anti-β-Actin antibody (clone AC-74, Sigma Aldrich, Gillingham, UK). Secondary antibodies were anti-rabbit or anti-mouse IgG HRP-linked (polyclonal, Cell Signalling). Blots were incubated with SuperSignal West Dura substrate (Thermo), developed with Amersham Hyperfilm (GE Healthcare, Chalfton, UK) or a Biorad gel imager and analysed using ImageJ or ImageLab (Biorad). The semiquantitative densitometric analysis, dependent on detection method, exposure-time and substrate reaction kinetics, does not imply linearity of the measurements. The resulting expression values support the visual representation of bands to avoid ambiguity.

### 4.7. Tube Formation Assay

A total of 2 × 10^4^ HUVECs in 100 µL growth medium or conditioned media were plated onto 96-well plates pre-coated with 70 µL of growth-factor-reduced Matrigel (Corning, Wiesbaden, Germany). Tube formation was stopped four to six hours after seeding, stained with 5 µM CFSE (Thermo) for 5 min, rinsed once with phosphate-buffered saline, and fixed for 20 min with 10% normal-buffered formalin. Wells were imaged at five different focal planes with the Nikon TE fluoresce microscope. Focal planes were merged using the multi-stack registration plugin for Fiji [61] and analysed using WimTube, available from https://www.wimasis.com/en/products/13/WimTube.

### 4.8. T-Cell Activation Assay

Lymphocytes were isolated from human peripheral blood from anonymised healthy donors (NHS Blood and Transfusion Service, Oxford, UK) as previously described [62]. A pan T-cell isolation kit (Miltenyi Biotec, Surrey, UK) was used to purify CD3-positive lymphocytes according to the manufacturer’s instructions. A total of 1.5 × 10^4^ DLD-1 cancer cells per well were placed in 96-well plates. After overnight incubation, 7.5 × 10^4^ CD3-positive T-cells per well were added and treated with or without 1 µL Human T-Activator CD3/CD28 Dynabeads (Thermo, Abingdon, UK) in 140 µL of cleared conditioned medium. T-cells were harvested 24 h later and stained with a Live/Dead Fixable Near-IR stain (Thermo, Abingdon, UK) and anti-CD4 (clone OKT4), anti-CD8 (clone HIT8a) and anti-CD25 (clone BC96) antibodies, all acquired from Biolegend (London, UK). Samples were measured using the AttuneNxT Flow Cytometer and analysed using FlowJo (Ashland, OR, USA).

### 4.9. Reverse Transcription Quantitative PCR (RT-qPCR)

Total RNA was extracted using the RNeasy Mini kit including an on-column DNA digest (Qiagen, Manchester, UK). From 500 to 800 ng total RNA was converted into cDNA using the QuantiTech-RT kit (Qiagen, Manchester, UK). A total of 10 μg of cDNA was used to detect sequence-specific amplicons using the primer sets summarised in Table 1. qPCR reactions were carried out with the SyGreen Blue Hi-ROX master mix (PCRBiosystems, Highgate, UK) and run in a StepOnePlusTM Real-time PCR system. Cycling conditions were: 95 °C for 2 min, and 40 cycles of denaturation at 95 °C for 5 s and annealing at 60 °C for 30 s. Gene expression relative to 18S RNA was determined using the Pfaffl method that incorporates the amplicon specific PCR efficiencies (E) estimated by cDNA dilution series [63] (1):

(1) $$Fold\text{-}change=\frac{E_{target}{}^{∆Ct\;target\;\left(Control-Sample\right)}}{E_{reference\;\left(18S\right)}{}^{∆Ct\;reference\;\left(Control-Sample\right)}}$$

### 4.10. Abdominal Imaging Window Implantation

This procedure was based on a previously described method [64]. CD17-SCID mice were prepared in a surgical unit, administered with inhalational anaesthesia and pre-operative aesthetic. Body temperature and respiration rates were monitored throughout the procedure. A one-cm cut was made along the abdominal midline approximately five mm underneath the sternum followed by blunt dissection around the cut to separate the connective tissue from the skin. The edge of a custom-made titanium imaging window frame (Workshop at the Department of Oncology, Oxford University) was fitted underneath the skin. Continuous sutures were used to secure the skin around the window frame, finishing with an intradermal suture. Approximately 2.5 × 10^5^ DLD-1 cells stably expressing mBeRFP (Figure S13) in 5 µL containing 30% of Matrigel and 1% of Evan’s blue dye were injected under the connective tissue and above the abdominal muscle layer. The chamber was then flushed with water to lyse non-injected cells by osmotic shock, tapped dry with sterile cotton swabs and flooded with saline. An 11-cm cover glass glued on the chamber’s lid was screwed onto the window frame. The animals were then placed onto a heat mat for post-operative recovery, and their health and tumour growth was monitored by visual examination. Animals with tumours covering at least 20% of the chamber were administered with 4 × 10^10^ EnAd-SA-dUnaG particles via the tail vein in two doses on days one and three. For validation, tumours were harvested ten minutes after the IV injection of an anti-CD31 PE-conjugated antibody (Biolegend). Cryosections were then stained by immunohistochemistry and imaged directly with the 710 Zeiss confocal microscope using 488 nm excitation and a spectral detector.

### 4.11. Intravital Two-Photon Imaging

Mice were imaged with a Zeiss LSM 880 microscope equipped with an aesthetic vaporiser and respiratory monitoring system. Stage and atmosphere were heated to 37 °C. To label perfused vessels, a Quatum dot-705 solution (1 µM, Invitrogen) was infused intravenously using a motorised pump at a rate of 0.84 µL·min^−1^. A mode-locked MaiTai laser tuned to 910 nm was used to simultaneously excite mBeRFP, UnaG and Qdot705. The Qdot705 signal was acquired through a BP700/100 filter with a non-descanned detector. GaAsP detectors were used to acquire the signal of mBeRFP selected by a BP 650/45 filter and the UnaG selected by a BP525/50 filter. On occasion, the second harmonic generation signal was registered with a 436/20 bandpass filter. Images were acquired in Z-stack tile scans with a pixel size of 0.823 µm and an image size per tile of 512 × 512 × 5 in x, y and z, respectively. A water immersion 20× objective made for UV-VIS-IR transmission with a numerical aperture of 1 was used.

### 4.12. Perfused Vessel Measurement

Z-stack tile scans from two-photon images were merged using the Zeiss Zen Black software. The relative perfused vascular volume of a non-infected tumour region and a region containing an established infection focus was calculated from 3D reconstructed in vivo images using the IMARIS (Bitplane, Zürich, Switzerland) volume plugin.

### 4.13. Ethics and Permission for Experimentation with Animals and Human Material

All animal experiments were conducted in accordance with the UK Home Office guidelines and the Animals (Scientific Procedures) Act 1989 and the UKCCCR Guidelines for the Welfare of Animals. Studies were approved by the University of Oxford Animal Welfare and Ethical Review Body (AWERB) under the project license 30/3391 (approved on 16 March 2016). Human blood was used as reviewed by the Research Ethical Committee under the institutional agreement R61446/RE001.

### 4.14. Statistics

Statistical analysis was carried out using GraphPad Prism 6.0 (GraphPad Software Inc., San Diego, CA, USA). Two-way ANOVA with Tukey post hoc testing was used to compare the effect of hypoxia and virus infection on gene expression, tube formation and T-cell activation assays. A non-parametric test was employed to analysed vascular volume derived from the intravital imaging study. Individual data points and means were plotted. The experimental error was shown as standard deviation, standard error of the mean or 95% confidence interval depending on the experiment. Significance levels were shown for * *p* ≤ 0.05, ** *p* ≤ 0.01, *** *p* ≤ 0.001, **** *p* ≤ 0.0001).

## 5. Conclusions

Taken together, this study highlights the importance of the interplay between virus infection and the surrounding microenvironment. The implications of the attenuated HIF signalling and low vascular perfusion observed in adenovirus-infected tumour nodules after systemic delivery may impact virus dissemination, alleviate immunosuppression, aggravate hypoxia, or perhaps even boost cytotoxicity. Elucidating these questions is crucial to understanding the anti-cancer mode of action of oncolytic adenovirus in a wider context and imperative to effectively dose and time companion drugs or arm adenovirus platforms.

## Figures and Tables

**Table 1:** Primer sequences, concentration and PCR efficiencies.

Gene	Primer Set (5′ → 3′)		Primer Concentration	PCR Efficiency	NCBI Accession
*18S*	Forward	GCCCGAAGCGTTTACTTTGA	100 nM	1.938	NR_145819
	Reverse	TCCATTATTCCTAGCTGCGGTATC			
*HIF-1α*	Forward	TTCACCTGAGCCTAATAGTCC	300 nM	1.944	NM_001530
	Reverse	CAAGTCTAAATCTGTGTCCTG			
*HIF-2α*	Forward	GAGACGGAGGTGTTCTATG	400 nm	1.970	NM_001430
	Reverse	TTCAGAGCAAACTGAGGAG			
*GLUT1*	Forward	ATACTCATGACCATCGCGCTAG	300 nM	2.010	NM_006516
	Reverse	AAAGAAGGCCACAAAGCCAAAG			
*VEGF*	Forward	CTACCTCCACCATGCCAAGT	500 nM	1.930	NM_001025368
	Reverse	CTCGATTGGATGGCAGTAGC			
*PCNA*	Forward	GTGTTGGAGGCACTCAAGG	400 nm	2.050	NM_182649.2
	Reverse	TGAGCTGCACCAAAGAGACG			
*TBP*	Forward	CTCCCGGAATCCCTATCTT	400 nm	2.061	NM_001172085.1
	Reverse	GCCTTTGTTGCTCTTCCA			

## Supplementary Materials

The following are available online at https://www.mdpi.com/2072-6694/12/4/851/s1, Figure S1: Tracking of virus persistence in tumour xenografts by bioluminescence imaging, Figure S2: Controls for immunohistochemistry analysis of Figure 1 and Appendix A Figure A1, Figure S3: Uncropped scans of western blots found in Figure 1D, Figure S4: Uncropped scans of western blots found in Figure 1E, Figure S5: HIF-1α down-regulation upon virus infection is virus-dose dependent, Figure S6: Uncropped scans of western blots in Figure 2, Figure S7: Down-regulation of HIF-1α is independent of proteasomal, PHD, and VHL activity but dependent on mRNA availability in HCT116 cells, Figure S8: Uncropped scans of western blots in Figure S7, Figure S9: Uncropped scans of western blots in Figure 3, Figure S10: Filtration of conditioned media from infected tumour cells by a 100 kDa molecular weight cut-off filter depletes live virus particles, Figure S11: Gating strategy for T-cell activation assay, Figure S12:1 Generation of EnAd encoding a destabilised UnaG (dUnaG) under the major late promoter, Figure S13: Generation of a mBeRFP expressing DLD-1 cell line for two-photon imaging.
