# Supplementary material for: Attenuation of the Hypoxia Inducible Factor Pathway after Oncolytic Adenovirus Infection Coincides with Decreased Vessel Perfusion
Source: Cancers (Basel). 2020 Apr 1;12(4):851. doi: 10.3390/cancers12040851 (PMC7225929; doi:10.3390/cancers12040851)
Supplement: Supplementary file 1 [file cancers-12-00851-s001.pdf]

Supplementary Materials: Attenuation of the Hypoxia Inducible Factor Pathway after Oncolytic Adenovirus Infection Coincides with Decreased Vessel Perfusion

Iris Yousaf, Jakob Kaeppler, Sally Frost, Len W. Seymour and Egon J. Jacobus

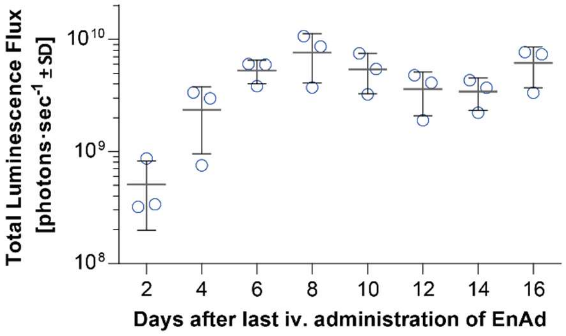

Figure S1. Tracking of virus persistence in tumour xenografts by bioluminescence imaging.Xenograft tumours used for histological analysis in Figure1 were tracked by bioluminescence to show the persistence of EnAd infection in these tumours over 16 days after virus infection (n = 3). 150 mg·kg^{-1} of a D-luciferin solution (#LUCK-100, GoldBio) was injected subcutaneously in the dorsocervical area. Ten minutes later the mice were anaesthetised with isoflurane and placed into a heat and respiration-controlled chamber of the IVIS Spectrum (Perkin Elmer). Images were acquired every minute for a maximum of 30 min to obtain a kinetic sequence. The total photon flux (photons per second per square centimetre) was calculated for every image and mouse in the sequence deducting the average background value. The images corresponding to the ≥94 % of the maximal luminescence intensity during kinetic measurement were compared. This ensured an accurate measurement of virus transduction when comparing acquisitions of the same animals at different time points.

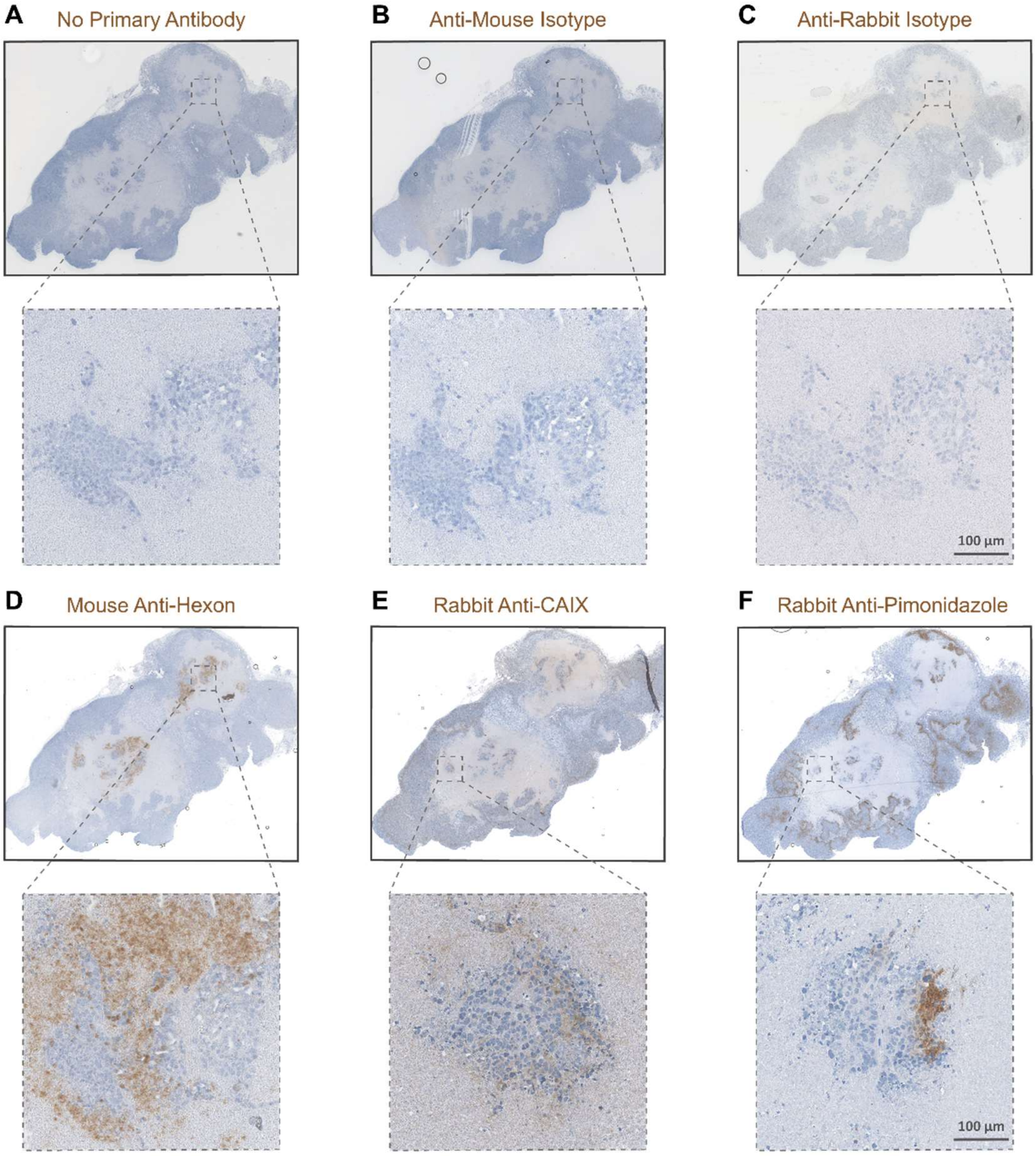

Figure S2. Controls for immunohistochemistry analysis in Figure 1 and Appendix Figure A1. Serial sections were stained with either no primary antibody (**A**), mouse isotype control (**B**), rabbit isotype control (**C**), mouse anti-Hexon antibody (**D**), rabbit anti-CAIX (**E**) or rabbit anti-pimonidazole antibody (**F**). All slides were counter-stained with haematoxylin.

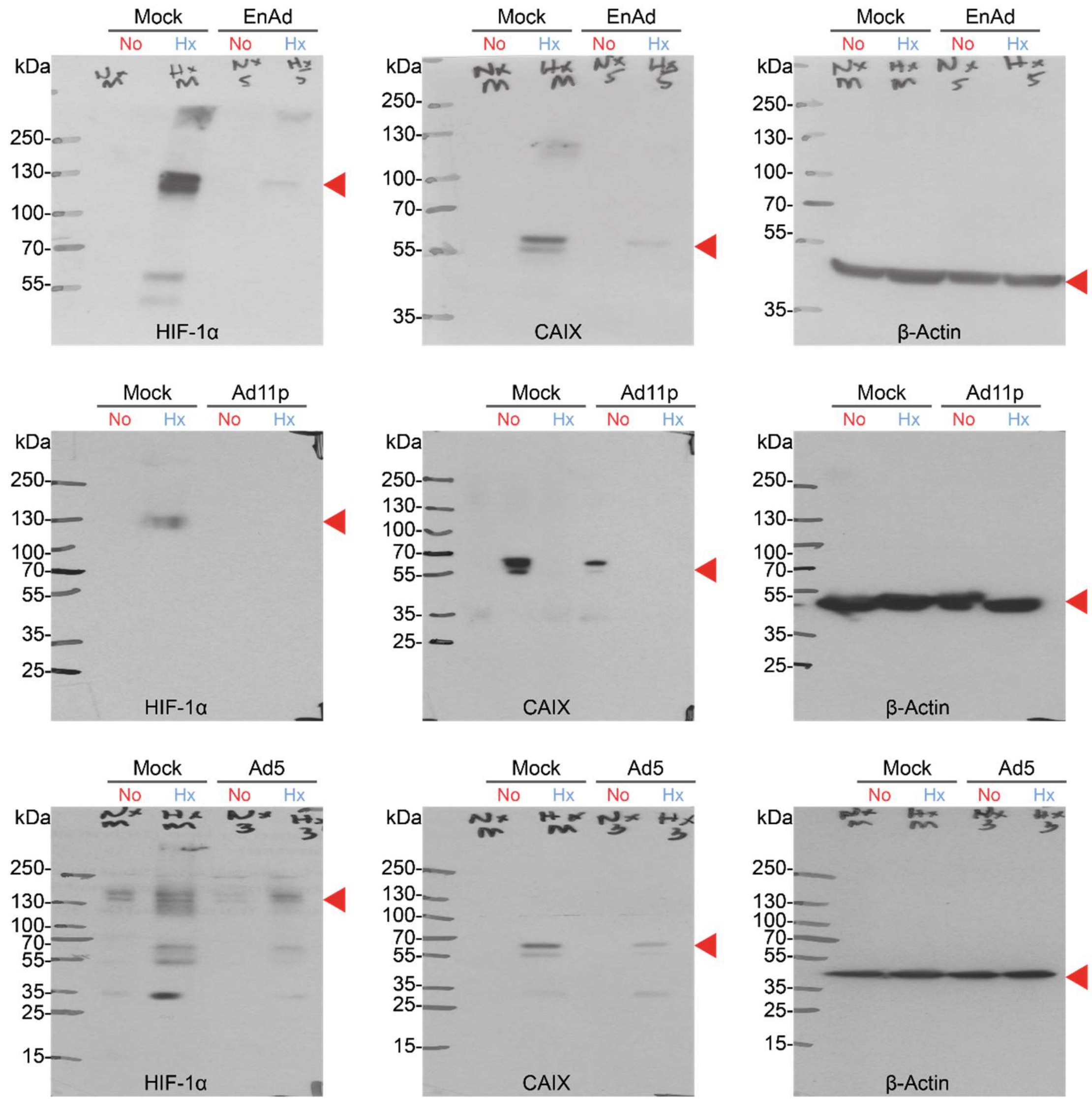

Figure S3. Uncropped scans of western blots found in Figure 1D.

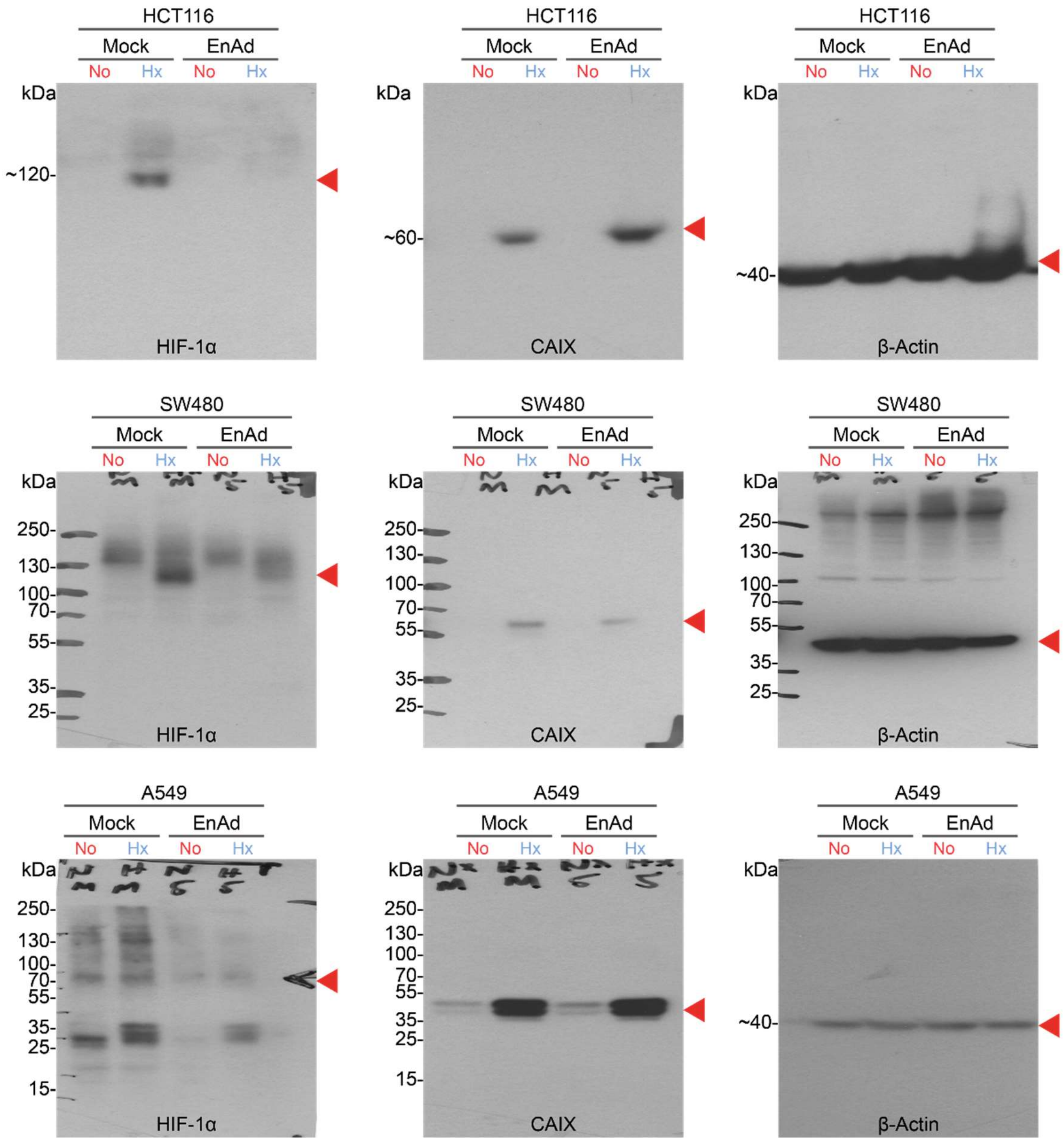

Figure S4. Uncropped scans of western blots found in Figure 1E.

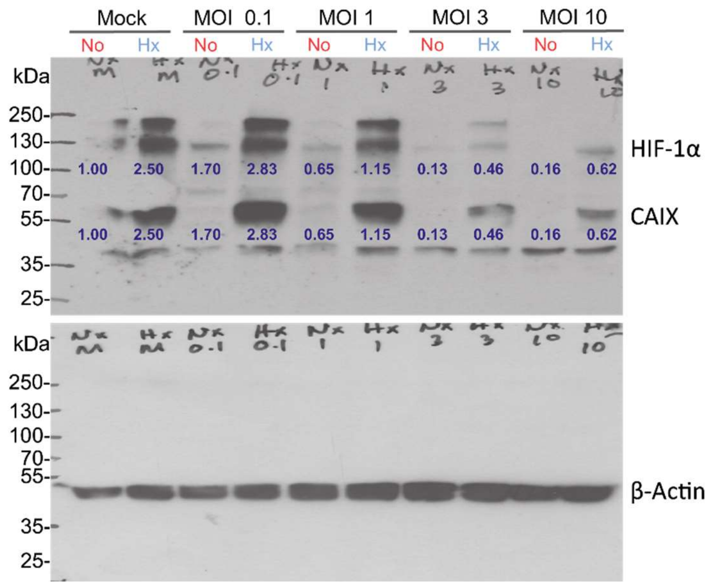

Figure S5. HIF-1α down-regulation upon virus infection is virus-dose dependent. DLD-1 cells were pre-exposed to hypoxia or normoxia for 18 h and infected with EnAd at multiplicity of infection 0.1, 1, 3 and 10. The infection continued in hypoxia or normoxia for a further 24 h before Western blot analysis.

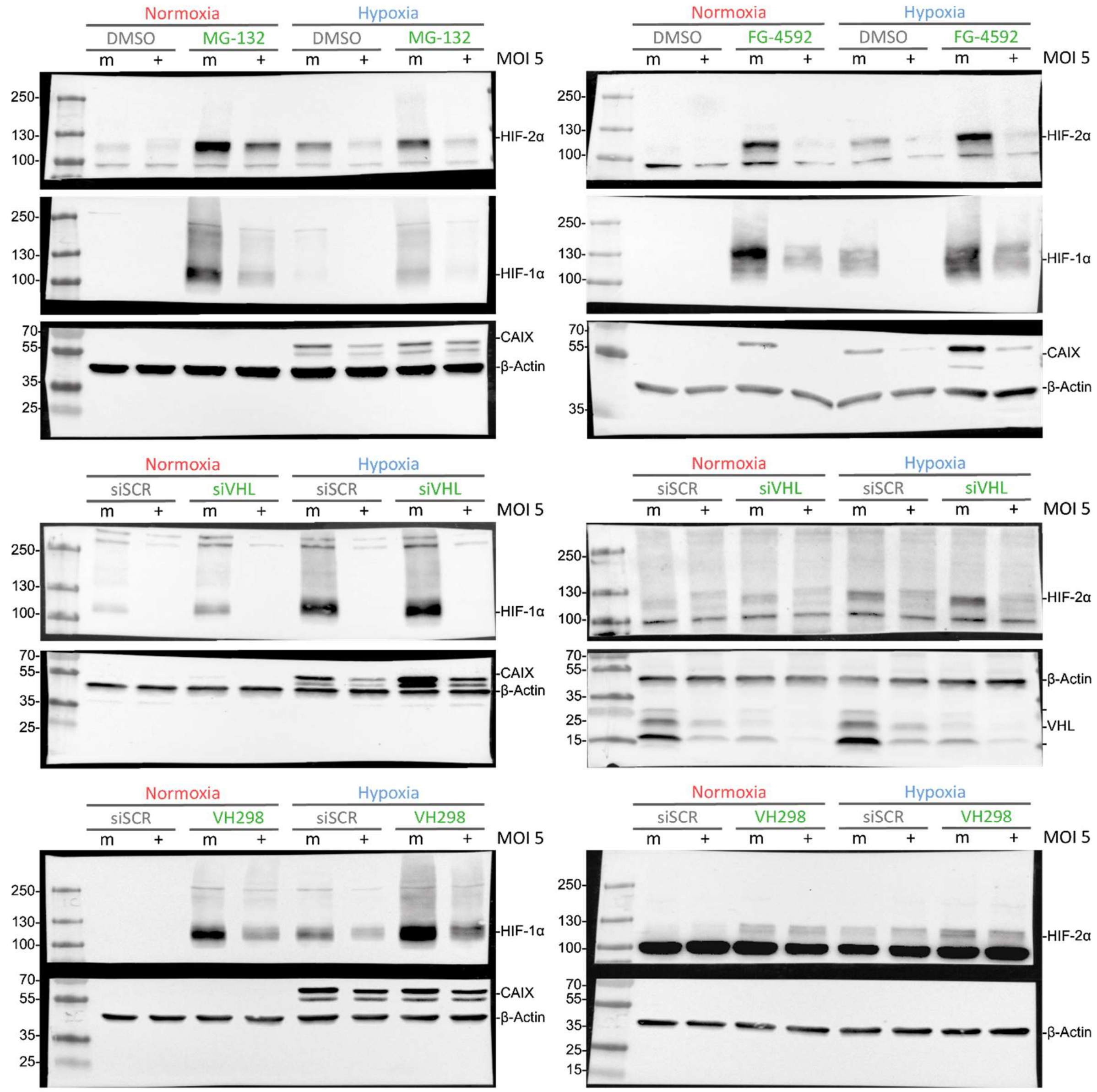

Figure S6. Uncropped scans of western blots in Figure 2.

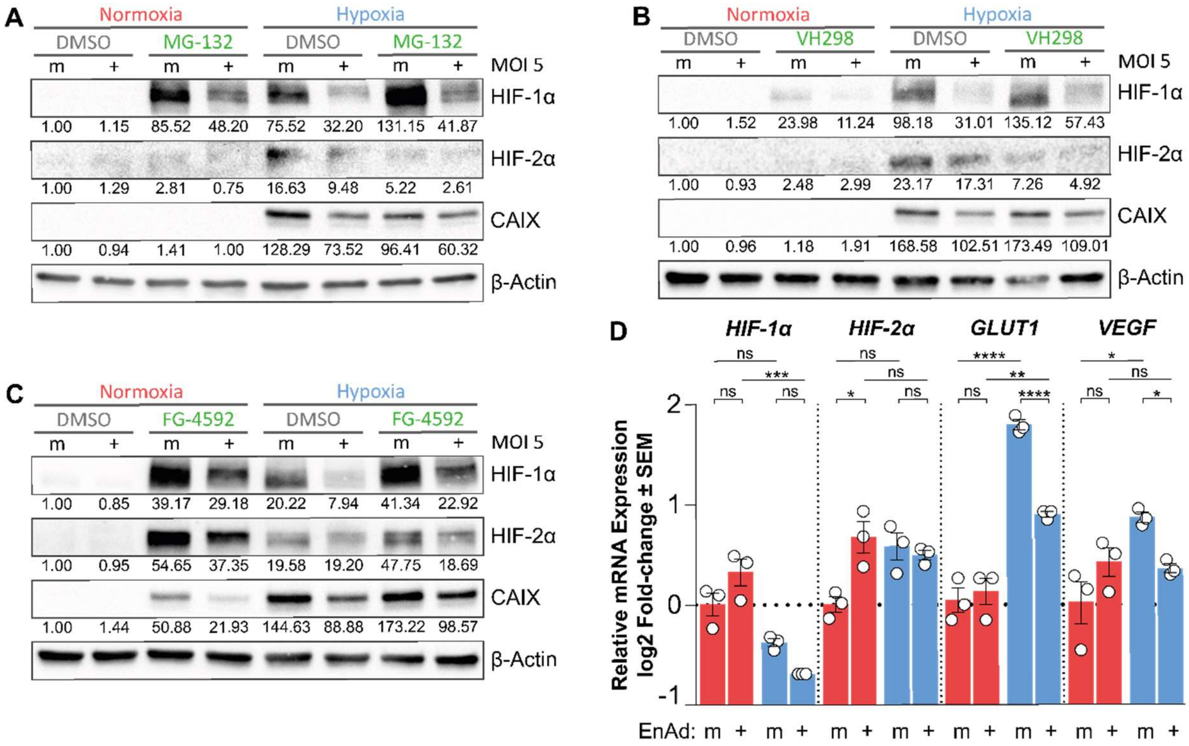

Figure S7. Down-regulation of HIF-1*α* is independent of proteasomal, PHD and VHL activity but dependent on mRNA availability in HCT116 cells. (**A**) HCT116 cells were pre-exposed to hypoxia (blue) or normoxia (red) for 18 h and infected with EnAd at MOI of 5 The infection continued in hypoxia or normoxia for a further 24 h before Western blot analysis. Proteasomal inhibition was achieved by adding 5 μM MG-132 six hours before harvest. (**B**) Inhibition of VHL was achieved VH298 treatment (100 μM) eight hours before harvest. (**C**) PHD inhibition was achieved by adding 75 μM FG-4592 two hours after infection. (**D**) Using the same hypoxia exposure, the mRNA expression of *HIF-1α, HIF-2α, GLUT1* and *VEGF* was measured by RT-qPCR 24 h after infection, (m: mock, +: infected, $n = 3$, ANOVA with Tukey post hoc test, ns: not significant, * $p \leq 0.05$, ** $p \leq 0.01$, *** $p \leq 0.001$, **** $p \leq 0.0001$).

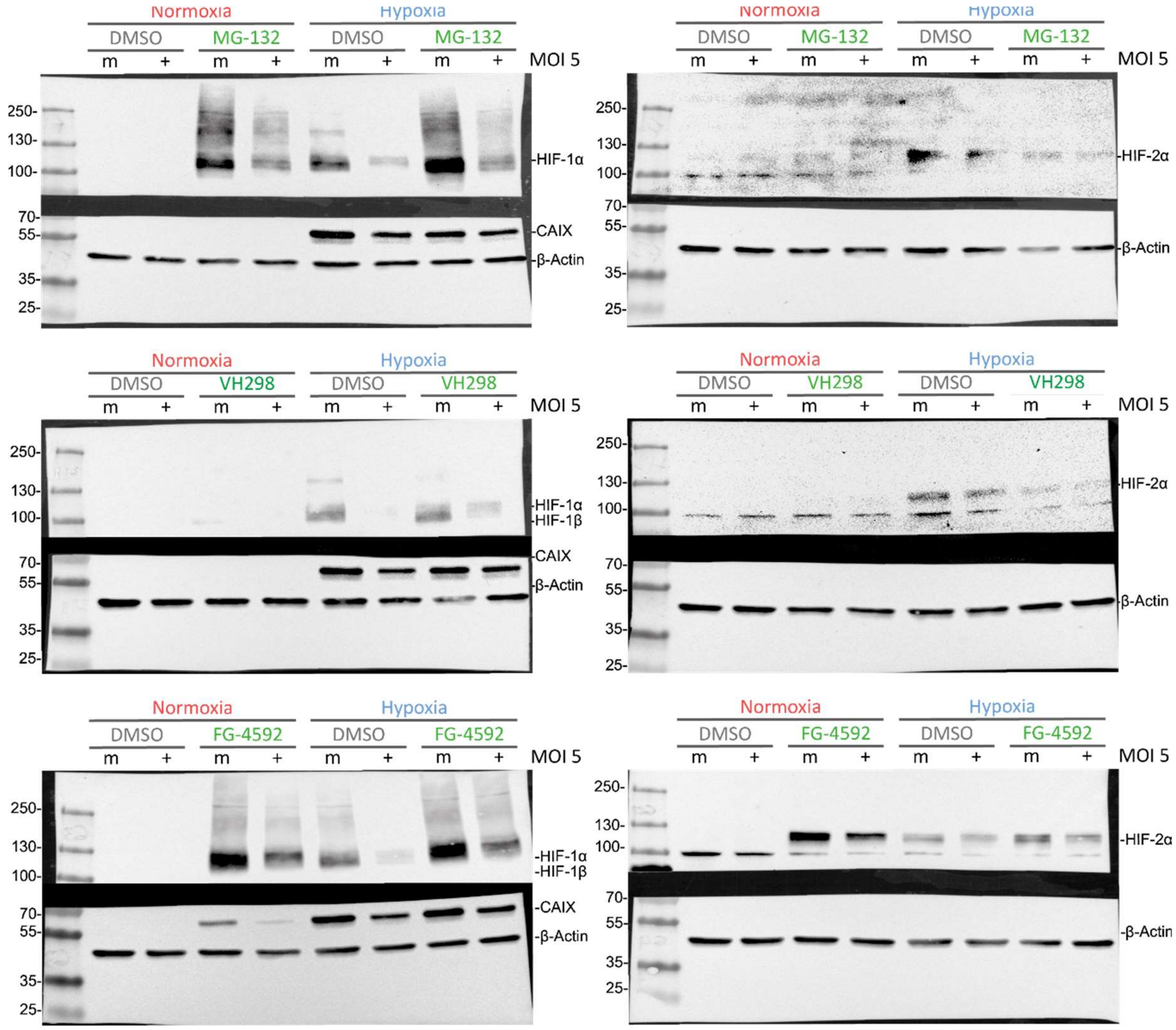

Figure S8. Uncropped scans of western blots in Figure S7.

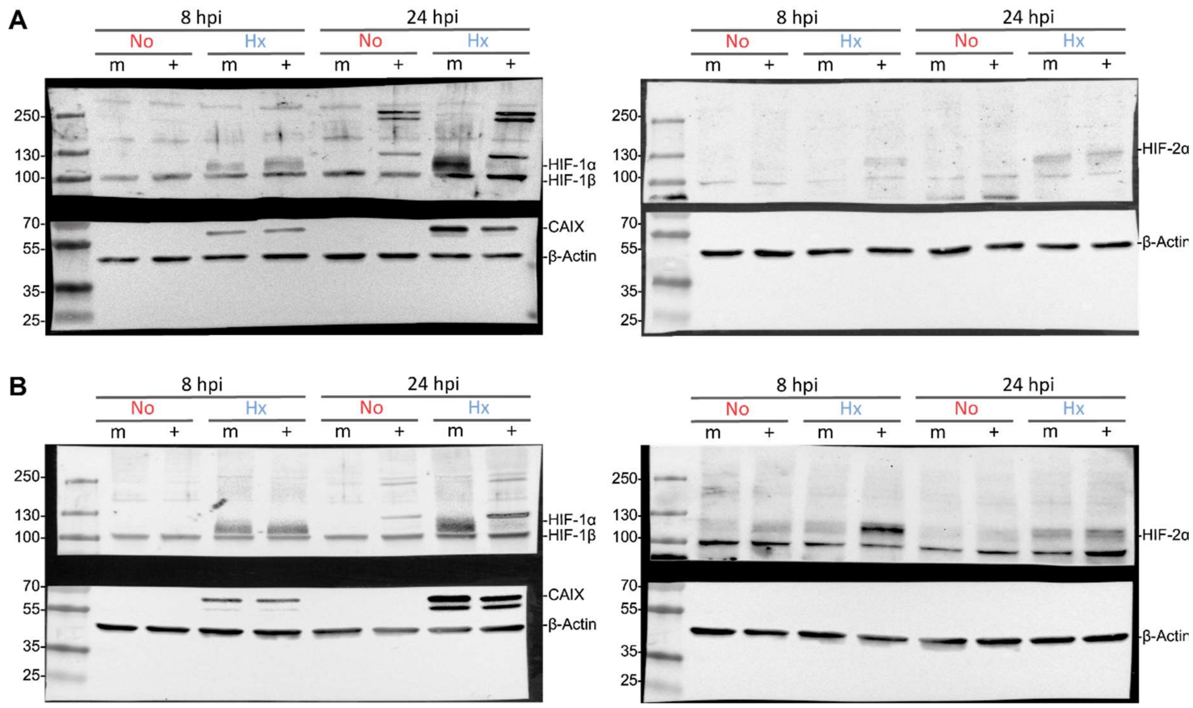

Figure S9. Uncropped scans of western blots in Figure 3.(**A**) DLD-1 and (**B**) HCT116.

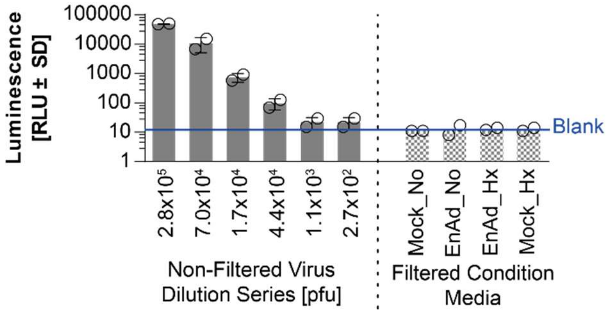

Figure S10. Filtration of conditioned media from infected tumour cells by a 100 kDa molecular weight cut-off filter depletes live virus particles. Infection of DLD-1 cells with an MOI gradient led to robust and dose-dependent luminescence at 24 h post-infection (280 000-270 plaque-forming units-pfu). Conversely, infection with conditioned media harvested for Figure 4 was passed through a 100 kDa molecular weight cut-off centrifugal unit and luminescence values are indistinguishable from the blank.

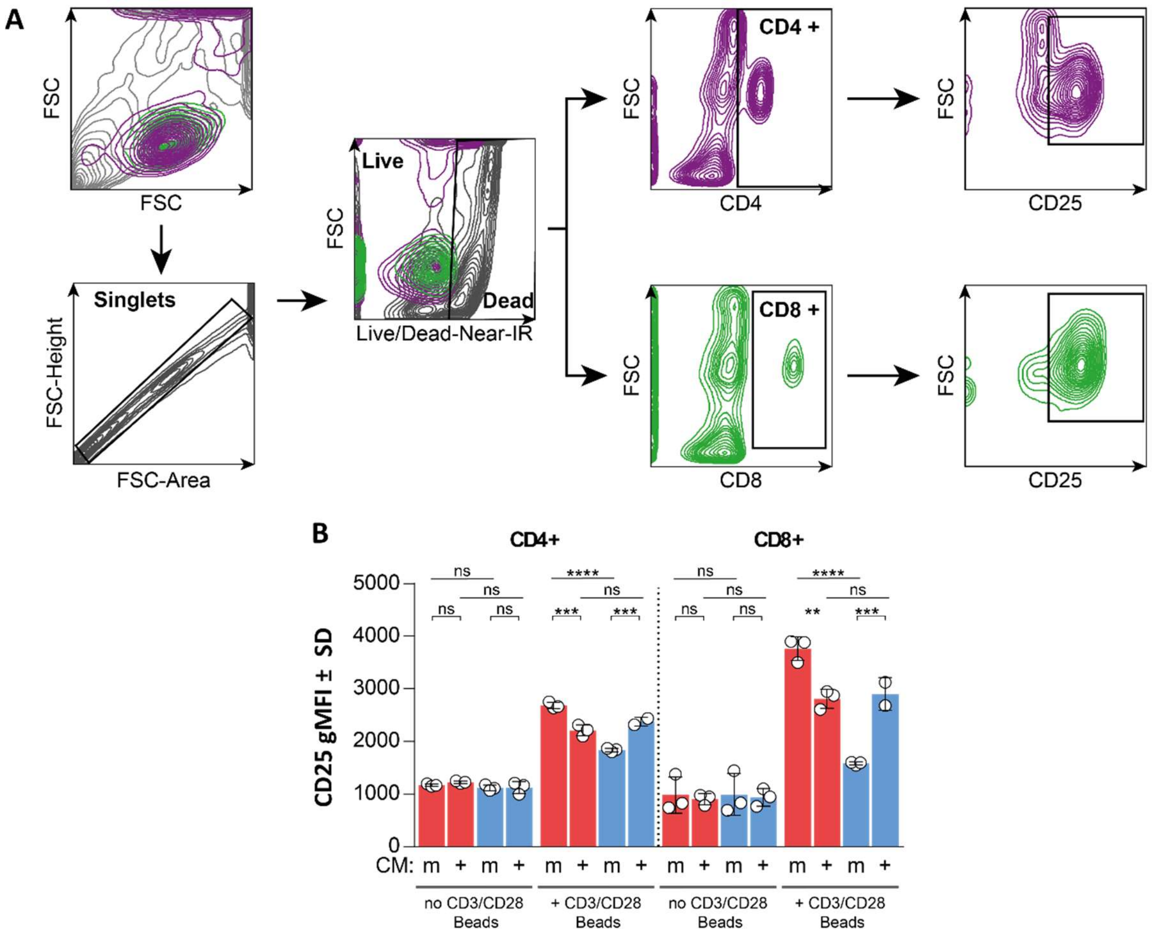

Figure S11. Gating strategy for T-cell activation assay. (**A**) Singlets were selected based on the forward-scatter area and height plot. Dead cells were discriminated with the Live/Dead-Near-IR stain. CD25 was measured within CD8 and CD4 positive populations. (**B**) CD25 geometric mean fluorescence intensity of CD4 and CD8 populations in co-cultures treated with conditioned media in the presence or absence of CD3/CD28 activation beads (as in Figure 4, hypoxia is red, and blue is normoxia). Statistical significance was tested by two-way ANOVA with Tukey post hoc test, $n = 3$, ns: not significant, * $p \leq 0.05$, ** $p \leq 0.01$, *** $p \leq 0.001$, **** $p \leq 0.0001$.

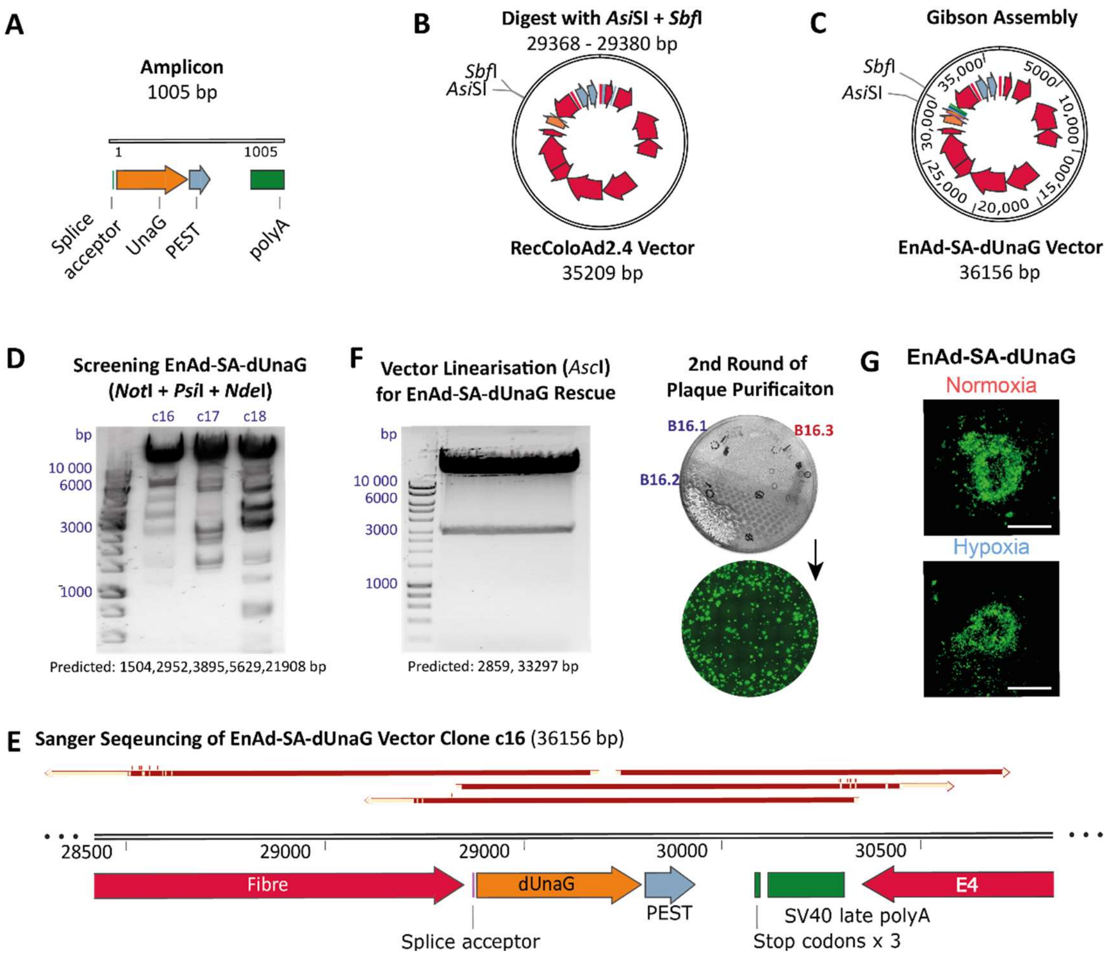

Figure S12. Generation of EnAd encoding a destabilised UnaG (dUnaG) under the major late promoter. (**A**) A UnaG construct was synthesized *de novo* containing a splice acceptor on the 5′ end, and a destabilizing PEST signal and a polyA-tail on the 3′ end. This construct was amplified with primers introducing homology arms to EnAd at the *Asi*SI and *Sbf*I sites. (B) RecColoAd2.4 backbone was digested with *Asi*SI and *Sbf*I. (**C**) Backbone and amplicon were ligated using Gibson assembly and transformed in DH10β cells. (**D**) Colonies were screened by restriction fragment analysis using *Not*I, *Psi*I and *Nde*I, clones 16, 17 and 18 are shown. (**E**) Correct insertion of splice acceptor-UnaG-PEST-polyA cassette between Fibre and E4 genes was confirmed by Sanger sequencing. (**F**) Clone 16 was linearised using *Asc*I enzyme removing the bacterial-derived sequences before transfection into cells. The rescued virus was plaque purified twice. Clone B16.3 was selected, propagated, and purified. The fluorescence EnAd-SA-dUnaG clone 16.3 was visualized during a plaque assay. (**G**) The oxygen-independent fluorescence was confirmed during a plaque assay performed in normoxia and hypoxia for four days.

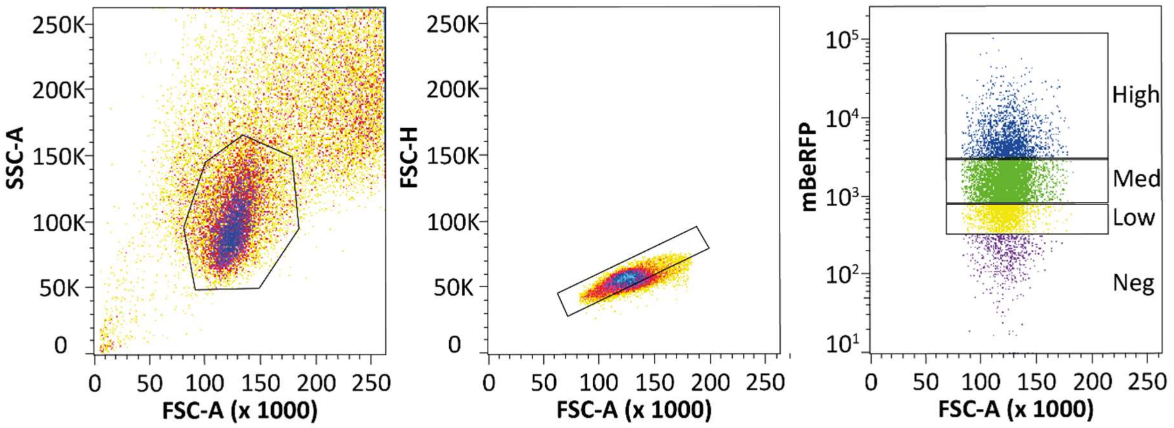

Figure S13. Generation of a mBeRFP expressing DLD-1 cell line for two-photon imaging. DLD-1 cells stably transduced with lentivirus encoding CMV-mBeRFP were sorted according to their expression level using fluorescence-activated cell sorting (FACS) at 488 nm excitation. The population with high levels of mBeRFP expression was isolated and used for intravital imaging in Figure 5.

© 2020 by the authors. Licensee MDPI, Basel, Switzerland. This article is an open access article distributed under the terms and conditions of the Creative Commons Attribution (CC BY) license (http://creativecommons.org/licenses/by/4.0/).
